# Burden of chronic kidney disease and its risk-attributable burden in 137 low-and middle-income countries, 1990–2019: results from the global burden of disease study 2019

**DOI:** 10.1186/s12882-021-02597-3

**Published:** 2022-01-05

**Authors:** Changrong Ke, Juanjuan Liang, Mi Liu, Shiwei Liu, Chunping Wang

**Affiliations:** 1 School of Public Health, Weifang Medical University, 261053 Weifang, China; 2 Chinese Center for Disease Control and Prevention, Beijing, 102206 China

**Keywords:** Chronic kidney disease, Burden of disease, Disability-adjusted life years, Population attributable faction, Risk factors

## Abstract

**Background:**

Chronic kidney disease (CKD) is a global public health concern, but its disease burden and risk-attributable burden in CKD has been poorly studied in low - and middle-income countries (LMICs). This study aimed to estimate CKD burden and risk-attributable burden in LMICs from 1990 to 2019.

**Methods:**

Data were collected from the Global Burden of Disease (GBD) Study 2019, which measure CKD burden using the years lived with disability (YLDs), years of life lost (YLLs), disability-adjusted life-years (DALYs) and calculate percentage contributions of risk factors to age-standardized CKD DALY using population attributable fraction (PAF) from 1990 to 2019. Trends of disease burden between 1990 and 2019 were evaluated using average annual percent change (AAPC). The 95% uncertainty interval (UI) were calculated and reported for YLDs, YLLs, DALYs and PAF.

**Results:**

In 2019, LICs had the highest age-standardized DALY rate at 692.25 per 100,000 people (95%UI: 605.14 to 785.67), followed by Lower MICs (684.72% (95%UI: 623.56 to 746.12)), Upper MICs (447.55% (95%UI: 405.38 to 493.01)). The age-standardized YLL rate was much higher than the YLD rate in various income regions. From 1990 to 2019, the age-standardized DALY rate showed a 13.70% reduction in LICs (AAPC = -0.5, 95%UI: − 0.6 to − 0.5, *P* < 0.001), 3.72% increment in Lower MICs (AAPC = 0.2, 95%UI: 0.0 to 0.3, *P* < 0.05). Age-standardized YLD rate was higher in females than in males, whereas age-standardized rates of YLL and DALY of CKD were all higher in males than in females in globally and LMICs. Additionally, the YLD, YLL and DALY rates of CKD increased with age, which were higher in aged≥70 years in various income regions. In 2019, high systolic blood pressure, high fasting plasma glucose, and high body-mass index remained the major causes attributable age-standardized CKD DALY. From 1990 to 2019, there were upward trends in the PAF of age-standardized DALY contributions of high fasting plasma glucose, high systolic blood pressure, and high body-mass index in Global, LICs, Lower MICs and Upper MICs. The greatest increase in the PAF was high body-mass index, especially in Lower MICs (AAPC = 2.7, 95%UI: 2.7 to 2.8, *P* < 0.001). The PAF of age-standardized DALY for high systolic blood pressure increased the most in Upper MICs (AAPC = 0.6, 95%UI: 0.6 to 0.7, *P* < 0.001).

**Conclusions:**

CKD burden remains high in various income regions, especially in LICs and Lower MICs. More effective and targeted preventive policies and interventions aimed at mitigating preventable CKD burden and addressing risk factors are urgently needed, particularly in geographies with high or increasing burden.

**Supplementary Information:**

The online version contains supplementary material available at 10.1186/s12882-021-02597-3.

## Introduction

Chronic kidney disease (CKD) is a global public health concern [1], and a risk factor for adverse outcomes in other diseases [2, 3]. CKD can be defined as a sustained damage of renal parenchyma that leads to chronic deterioration of renal function and may gradually progress to end-stage renal disease (ESRD) [4]. Without kidney replacement therapy (dialysis or kidney transplantation), ESRD remains uniformly fatal [4]. Studies have shown that kidneys are linked to Coronavirus disease 2019 (COVID-19) infection [5]. COVID-19 has been declared as a global pandemic by the World Health Organization [5], and the incidence of acute kidney injury (AKI) in patients infected with COVID-19 is around 3–15% [6, 7]. AKI can cause ESRD directly, and increase the risk of developing incident CKD and worsening of underlying CKD [8]. According to the Global Burden of Disease (GBD) study, disability-adjusted life-years (DALYs) for CKD has risen from 29th in 1990 to 18th in 2019 [9]. The global burden of CKD is rapidly increasing and is projected to become the 5th most common cause of years of life lost worldwide by 2040 [10, 11], with the burden of CKD increasing faster in low - and middle-income countries (LMICs) [12]. The burden of CKD is studied predominantly in high-income countries, with few studies in LMICs, and even fewer studies on attributable disease burden [2]. In this study, we aimed to analyzed and compared the trends of disease burden of CKD as well as its attributable disease burden in LMICs from 1990 to 2019, by using the data from the GBD 2019 study.

## Methods

### Data sources

Data for this study were obtained from the GBD 2019 study. The GBD 2019 provides estimates of years lived with disability (YLDs), years of life lost (YLLs), and disability-adjusted life-years (DALYs) disaggregated due to 369 diseases and injuries, and 87 risk factors and combinations of risk factors in 204 countries and territories from 1990 to 2019 [9, 13]. Detailed methods of GBD 2019 have been published elsewhere [9, 13]. The GBD 2019 attributable burden estimates followed the general framework established for comparative risk assessment (CRA) [14, 15]. Compared with previous GBD studies [16–18], the GBD 2019 study developed new approaches to estimate risk exposure and risk-attributable burden by integrating all accessible data from multiple epidemiological studies in various countries [13]. These studies included high-quality systematic reviews, cohorts, trials, case-control studies and other observational studies [13].

This study adherence to the Guidelines for Accurate and Transparent Health Estimates Reporting (GATHER) recommendations [19]. All data used in this study were obtained from the Institute for Health Metrics and Evaluation (IHME) website [20].

In the GBD study, chronic kidney disease was recognized as a disease as well as a metabolic risk factor [21]. In this analysis, we treated chronic kidney disease as a disease. CKD was defined as eGFR (estimated glomerular filtration rate) of < 60 mL/min per 1.73 m^2^ [21].

### Definitions

We used years lived with disability (YLDs), years of life lost (YLLs) and disability-adjusted life-years (DALYs) to measure the burden caused by CKD. YLDs measures the amount of time people lose to diseases and injuries that degrade their level of health but do not cause death. YLLs is a measure of premature death within a group of people. DALYs is a comprehensive index to evaluate the disease burden of disability and premature death, it is calculated by adding the YLLs and the YLDs [22, 23].

Due to the potential effects of climate change on human health, two new risk factors (high and low non-optimal temperatures) have been added in GBD 2019 [13]. 7 risk factors, i.e. high temperature, low temperature, lead exposure, diet high in sodium, high systolic blood pressure, high fasting plasma glucose, high body mass index, were considered to be associated with CKD in GBD 2019.

In our analyses we classified countries according to World Bank Income Levels in 2019, a total of 137 low-and middle-income countries (LMICs) were categorized into three groups: 31 low-income countries (LICs), 47 lower-middle income countries (Lower MICs) and 59 upper-middle income countries (Upper MICs).

### Statistical analyses

We computed counts, and age-standardized rates (per 100,000) to quantify the burden of CKD. YLDs, YLLs and DALYs were metrics used to measure the burden of CKD and reported by specific region, year, sex, age. Age-standardized rates (per 100,000) were standardized by the global age-standard population. The percentage contributions of risk factors to age-standardized CKD DALY were calculated by the population attributable fraction (PAF). Percentage change was used to show the magnitude of the change in YLDs, YLLs, DALYs and PAF from 1990 to 2019. Trends of disease burden between 1990 and 2019 were evaluated using average annual percent change (AAPC), which were calculated by the Joinpoint Regression Program (Version 4.9.0.0. March 2021) [24]. We considered *P* < 0.05 to be significant.

### Uncertainty interval

For each estimated rate and number of YLDs, YLLs, DALYs, and PAF of risk-attributable CKD DALYs, we reported its 95% uncertainty interval (UI). 95% UI was calculated by taking 1000 draws from the posterior distribution of each quantity and using the 25th and 975th-ordered draw of the uncertainty distribution [25].

## Results

CKD-related YLDs, YLLs and DALYs increased to varying degrees in various income regions from 1990 to 2019 (Table 1). In 2019, LICs had the highest age-standardized DALY rate at 692.25 per 100,000 people (95%UI: 605.14 to 785.67) in 2019, followed by Lower MICs (684.72% (95%UI: 623.56 to 746.12)), Upper MICs (447.55% (95%UI: 405.38 to 493.01)), while HICs (292.93% (95%UI: (263.61 to 322.94)) had the lowest rate. The age-standardized YLL rate was much higher than the YLD rate at the global level and in various income regions. From 1990 to 2019, the age-standardized YLD rate all showed an upward trend in various income regions(*P* < 0.001), while YLL rate decreased in LICs (AAPC = − 0.7, 95%UI: − 0.7 to − 0.7), *P* < 0.001) and Upper MICs (AAPC = -0.3, 95%UI: − 0.4 to − 0.2, *P* < 0.001). The age-standardized DALY rate showed a 6.27% increment in Global (AAPC = 0.3, 95%UI: 0.2 to 0.4, *P* < 0.001), 12.75% increment in HICs (AAPC = 0.6, 95%UI: 0.5 to 0.7, *P* < 0.001), 3.72% increment in Lower MICs (AAPC = 0.2, 95%UI: 0.0 to 0.3, *P* < 0.05), whereas decreased 13.70% from 802.15 per 100,000 people (95%UI: 716.50 to 893.68) in 1990 to 692.25 per 100,000 people (95%UI: 605.14 to 785.67) in 2019 in LICs (AAPC = -0.5, 95%UI: − 0.6 to − 0.5, *P* < 0.001), and 2.25% reduction in Upper MICs (AAPC = 0.1, 95%UI: 0.0 to 0.1, *P* = 0.150) (Tables 2 and 3).

**Table 1:** Number of YLDs, YLLs, DALYs of CKD with percent change in specific region, 1990–2019

region	YLDs (in thousands)			YLLs (in thousands)			DALYs (in thousands)		
	1990	2019	% change	1990	2019	% change	1990	2019	% change
**Global**									
Male	1741.00(1257.88 to 2303.57)	4021.93(2903.65 to 5335.61)	131.01	9459.51(8850.56 to 10,238.32)	17,958.18(16,654.70 to 19,565.50)	89.84	11,200.51(10,402.02 to 12,151.42)	21,980.11(20,140.02 to 23,965.10)	96.24
Female	2136.55(1543.68 to 2754.34)	4724.57(3429.29 to 6179.89)	121.13	8167.50(7550.21 to 8778.44)	14,833.90(13,467.77 to 16,117.36)	81.62	10,304.06(9457.91 to 11,216.13)	19,558.48(17,699.94 to 21,467.98)	89.81
Both	3877.55(2790.33 to 5063.93)	8746.51(6320.23 to 11,508.24)	125.57	17,627.02(16,596.25 to 18,589.76)	32,792.08(30,450.91 to 35,049.54)	86.03	21,504.57(20,039.12 to 23,065.78)	41,538.59(38,291.81 to 45,037.86)	93.16
**HICs**									
Male	410.85(302.26 to 536.35)	814.79(598.87 to 1060.79)	98.32	1170.13(1137.44 to 1192.65)	2376.83(2222.72 to 2485.90)	103.13	1580.98(1463.78 to 1708.95)	3191.63(2927.57 to 3466.48)	101.88
Female	497.17(367.65 to 658.03)	884.67(656.84 to 1153.20)	77.94	1093.49(1030.47 to 1130.39)	2165.83(1892.11 to 2343.57)	98.07	1590.66(1446.26 to 1754.41)	3050.51(2714.86 to 3374.81)	91.78
Both	908.03(668.63 to 1190.11)	1699.47(1254.67 to 2213.26)	87.16	2263.62(2168.82 to 2317.97)	4542.67(4116.75 to 4812.88)	100.68	3171.64(2920.57 to 3460.03)	6242.13(5661.99 to 6839.95)	96.81
**LICs**									
Male	64.78(45.86 to 86.48)	183.03(128.83 to 243.54)	182.56	812.62(705.35 to 915.27)	1374.18(1155.57 to 1633.83)	69.11	877.40(771.03 to 981.89)	1557.22(1324.61 to 1816.35)	77.48
Female	86.82(62.37 to 114.16)	236.24(170.14 to 308.59)	172.11	725.37(579.46 to 886.26)	1123.96(956.11 to 1317.79)	54.95	812.19(666.47 to 978.52)	1360.20(1168.36 to 1567.74)	67.47
Both	151.60(108.49 to 199.48)	419.28(301.72 to 551.00)	176.58	1537.99(1323.56 to 1736.06)	2498.14(2131.37 to 2923.69)	62.43	1689.59(1467.73 to 1895.09)	2917.41(2511.73 to 3337.37)	72.67
**Lower MICs**									
Male	582.07(412.90 to 764.46)	1435.35(1022.53 to 1884.88)	146.59	4102.25(3652.28 to 4877.33)	8296.79(7396.62 to 9441.35)	102.25	4684.32(4232.22 to 5461.27)	9732.14(8727.25 to 10,944.03)	107.76
Female	679.46(485.04 to 888.82)	1716.13(1227.10 to 2237.84)	152.57	3402.13(2905.25 to 3892.33)	6549.77(5624.24 to 7307.56)	92.52	4081.59(3548.37 to 4611.32)	8265.90(7261.02 to 9217.41)	102.52
Both	1261.53(894.56 to 1645.02)	3151.48(2258.15 to 4109.39)	149.81	7504.37(6712.65 to 8256.48)	14,846.56(13,448.78 to 16,241.31)	97.84	8765.91(7935.09 to 9641.47)	17,998.04(16,344.91 to 19,659.97)	105.32
**Upper MICs**									
Male	682.27(491.68 to 914.77)	1586.01(1117.03 to 2119.58)	132.46	3368.40(3075.84 to 3669.85)	5897.67(5272.58 to 6534.90)	75.09	4050.67(3687.01 to 4402.69)	7483.69(6751.81 to 8336.16)	84.75
Female	871.74(631.51 to 1141.58)	1884.09(1367.00 to 2491.20)	116.13	2941.26(2679.18 to 3217.92)	4983.55(4468.38 to 5549.00)	69.44	3813.00(3424.11 to 4186.28)	6867.63(6115.64 to 7687.36)	80.11
Both	1554.01(1120.48 to 2050.69)	3470.10(2493.95 to 4600.83)	123.30	6309.66(5880.61 to 6755.34)	10,881.22(9964.84 to 11,836.99)	72.45	7863.67(7212.38 to 8502.03)	14,351.32(13,002.53 to 15,772.42)	82.50

*CKD* Chronic kidney disease, *YLD* Years lived with disability, *YLL* Years of life lost, *DALY* Disability-adjusted life-years, *HICs* High-income countries, *LICs* Low-income countries, *Lower MICs* Lower-middle income countries. *Upper MICs* Upper-middle income countries

**Table 2:** Age-standardized YLD, YLL and DALY rates of CKD with percent change in specific region, 1990–2019

region	Age-standardized YLD rate per 100,000 persons			Age-standardized YLL rate per 100,000 persons			Age-standardized DALY rate per 100,000 persons		
	1990	2019	% change	1990	2019	% change	1990	2019	% change
**Global**									
Male	83.06(60.14 to 108.94)	104.03(75.56 to 137.05)	25.25	454.55(426.04 to 488.90)	470.20(435.54 to 510.93)	3.44	537.61(499.52 to 580.01)	574.23(527.52 to 625.27)	6.81
Female	91.65(66.14 to 118.85)	113.13(81.95 to 147.68)	23.44	352.04(328.69 to 377.34)	350.49(318.55 to 380.64)	−0.44	443.69(409.36 to 481.56)	463.61(419.34 to 509.95)	4.49
Both	87.22(63.28 to 113.54)	108.40(78.51 to 142.56)	24.29	397.25(375.73 to 417.52)	406.46(377.87 to 434.14)	2.32	484.46(452.28 to 518.67)	514.86(474.91 to 558.86)	6.27
**HICs**									
Male	78.05(57.23 to 101.71)	92.63 (68.01 to 121.51)	18.67	227.74 (220.16 to 232.59)	243.88 (229.00 to 255.59)	7.09	305.79 (283.57 to 330.59)	336.51 (306.59 to 367.35)	10.04
Female	75.28(55.46 to 98.47)	87.77 (64.41 to 114.01)	16.60	152.35 (144.34 to 157.22)	169.67 (154.42 to 181.29)	11.37	227.63 (206.55 to 251.60)	257.44 (229.34 to 286.27)	13.10
Both	76.35(56.27 to 99.56)	89.85 (66.04 to 117.20)	17.69	183.46 (176.01 to 187.86)	203.08 (187.90 to 213.86)	10.69	259.81 (238.45 to 283.93)	292.93 (263.61 to 322.94)	12.75
**LICs**									
Male	61.50(44.10 to 80.97)	81.40(58.54 to 108.12)	32.35	834.31(734.05 to 920.96)	701.47(607.71 to 807.96)	−15.92	895.81(795.47 to 989.35)	782.87(682.59 to 899.47)	−12.61
Female	77.61(56.39 to 101.50)	97.51(71.57 to 126.69)	25.65	641.19(540.91 to 773.77)	518.89(450.09 to 600.83)	−19.07	718.80(614.24 to 852.54)	616.40(538.21 to 704.61)	−14.25
Both	69.97(50.87 to 91.78)	89.95(65.58 to 117.75)	28.56	732.18(651.61 to 822.89)	602.30(523.74 to 689.61)	−17.74	802.15(716.50 to 893.68)	692.25(605.14 to 785.67)	−13.70
**Lower MICs**									
Male	86.95(62.24 to 113.19)	108.85(78.37 to 142.55)	25.20	630.85(565.26 to 744.67)	651.12(579.51 to 739.73)	3.21	717.80(650.34 to 835.60)	759.97(681.95 to 856.46)	5.88
Female	97.80(70.30 to 127.70)	123.26(88.95 to 160.66)	26.04	508.15(441.45 to 572.10)	491.88(423.51 to 548.00)	−3.20	605.95(531.23 to 677.46)	615.15(541.46 to 684.47)	1.52
Both	92.29(66.32 to 120.32)	116.05(83.44 to 151.12)	25.75	567.85(514.72 to 621.02)	568.67(515.93 to 621.51)	0.14	660.14(601.55 to 723.28)	684.72(623.56 to 746.12)	3.72
**Upper MICs**									
Male	82.65(59.61 to 109.30)	104.87(74.89 to 140.33)	26.89	417.83(381.07 to 456.87)	391.98(351.77 to 433.27)	−6.19	500.48(456.39 to 545.26)	496.86(449.34 to 551.52)	−0.72
Female	95.10(68.56 to 124.85)	116.28(83.74 to 153.68)	22.28	332.04(302.90 to 362.72)	291.19(261.08 to 324.08)	−12.30	427.14(385.04 to 470.83)	407.47(361.69 to 454.97)	−4.61
Both	88.69(64.44 to 116.99)	110.37(79.29 to 146.64)	24.45	369.16(344.16 to 395.66)	337.18(308.47 to 366.80)	−8.66	457.84(419.92 to 494.42)	447.55(405.38 to 493.01)	−2.25

*CKD* Chronic kidney disease, *YLD* Years lived with disability, *YLL* Years of life lost, *DALY* Disability-adjusted life-years, *HICs* High-income countries, *LICs* Low-income countries, *Lower MICs* Lower-middle income countries, *Upper MICs* Upper-middle income countries

**Table 3:** The AAPC in age-standardized rates of YLD, YLL, and DALY for CKD,1990–2019

region	Age-standardized YLD rate per 100,000 persons		Age-standardized YLL rate per 100,000 persons		Age-standardized DALY rate per 100,000 persons	
	AAPC	*P*-Value	AAPC	*P*-Value	AAPC	*P*-Value
**Global**						
Male	1.1(1.0 to 1.3)	< 0.001	0.2(0.1 to 0.3)	< 0.001	0.3(0.3 to 0.4)	< 0.001
Female	1.1(1.0 to 1.2)	< 0.001	0.0 (−0.1 to 0.0)	0.330	0.2(0.1 to 0.3)	< 0.001
Both	1.1(1.0 to 1.2)	< 0.001	0.1(0.0 to 0.2)	0.006	0.3(0.2 to 0.4)	< 0.001
**HICs**						
Male	0.7(0.6 to 0.8)	< 0.001	0.4(0.4 to 0.5)	< 0.001	0.5(0.4 to 0.6)	< 0.001
Female	0.6(0.4 to 0.7)	< 0.001	0.6(0.5 to 0.6)	< 0.001	0.6(0.5 to 0.7)	< 0.001
Both	0.6(0.5 to 0.8)	< 0.001	0.6(0.5 to 0.6)	< 0.001	0.6(0.5 to 0.7)	< 0.001
**LICs**						
Male	1.0(0.9 to 1.1)	< 0.001	− 0.6(− 0.6 to − 0.6)	< 0.001	− 0.5(− 0.5 to − 0.5)	< 0.001
Female	0.8(0.7 to 0.9)	< 0.001	− 0.7(− 0.8 to − 0.7)	< 0.001	− 0.5(− 0.6 to − 0.5)	< 0.001
Both	0.9(0.8 to 1.0)	< 0.001	− 0.7(− 0.7 to − 0.7)	< 0.001	− 0.5(− 0.6 to − 0.5)	< 0.001
**Lower MICs**						
Male	1.1(0.9 to 1.3)	< 0.001	0.1(0.0 to 0.3)	0.054	0.3(0.1 to 0.4)	0.001
Female	1.0(0.9 to 1.1)	< 0.001	− 0.2(− 0.3 to − 0.1)	< 0.001	0.0 (0.0 to 0.1)	0.507
Both	1.1(0.9 to 1.2)	< 0.001	0.0 (− 0.1 to 0.1)	0.800	0.2(0.0 to 0.3)	0.006
**Upper MICs**						
Male	1.4(1.2 to 1.6)	< 0.001	− 0.2(− 0.2 to − 0.1)	< 0.001	0.1(0.1 to 0.2)	0.001
Female	1.3(1.1 to 1.5)	< 0.001	−0.5(− 0.6 to − 0.4)	< 0.001	0.0 (− 0.1 to 0.1)	0.443
Both	1.4(1.2 to 1.5)	< 0.001	− 0.3(− 0.4 to − 0.2)	< 0.001	0.1(0.0 to 0.1)	0.150

*CKD* Chronic kidney disease, *AAPC* average annual percentage change, *YLD* Years lived with disability, *YLL* Years of life lost, *DALY* Disability-adjusted life-years, *HICs* High-income countries, *LICs* Low-income countries, *Lower MICs* Lower-middle income countries, *Upper MICs* Upper-middle income countries

**Fig. 1:**
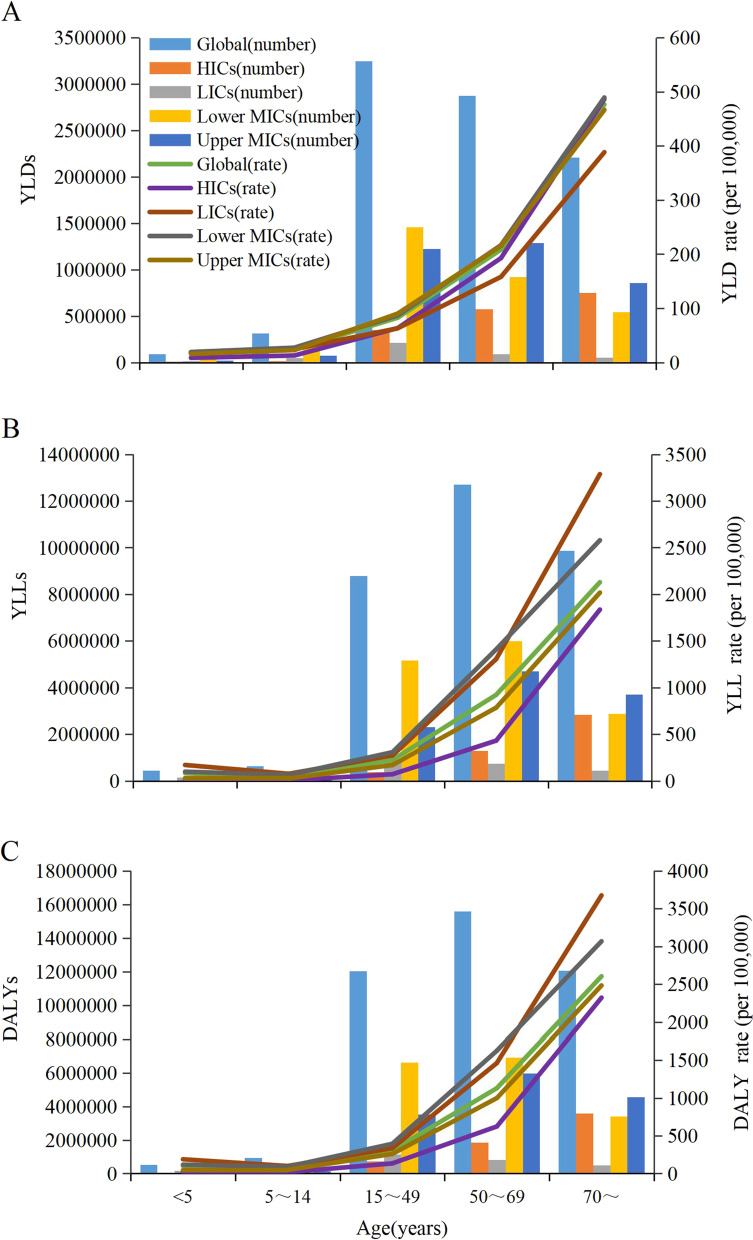
Age-specific numbers and rates of YLDs (**A**), YLLs (**B**) and DALYs (**C**) of CKD, 2019. YLD=Years lived with disability. YLL = Years of life lost. DALY=Disability-adjusted life-years

In terms of gender, age-standardized YLD rate was higher in females than in males, whereas age-standardized rates of YLL and DALY of CKD were all higher in males than in females in globally and LMICs (Table 2). Additionally, the YLD, YLL and DALY rates of CKD increased with age in various income regions, which were higher in aged≥70 years (Fig. 1).

**Fig. 2:**
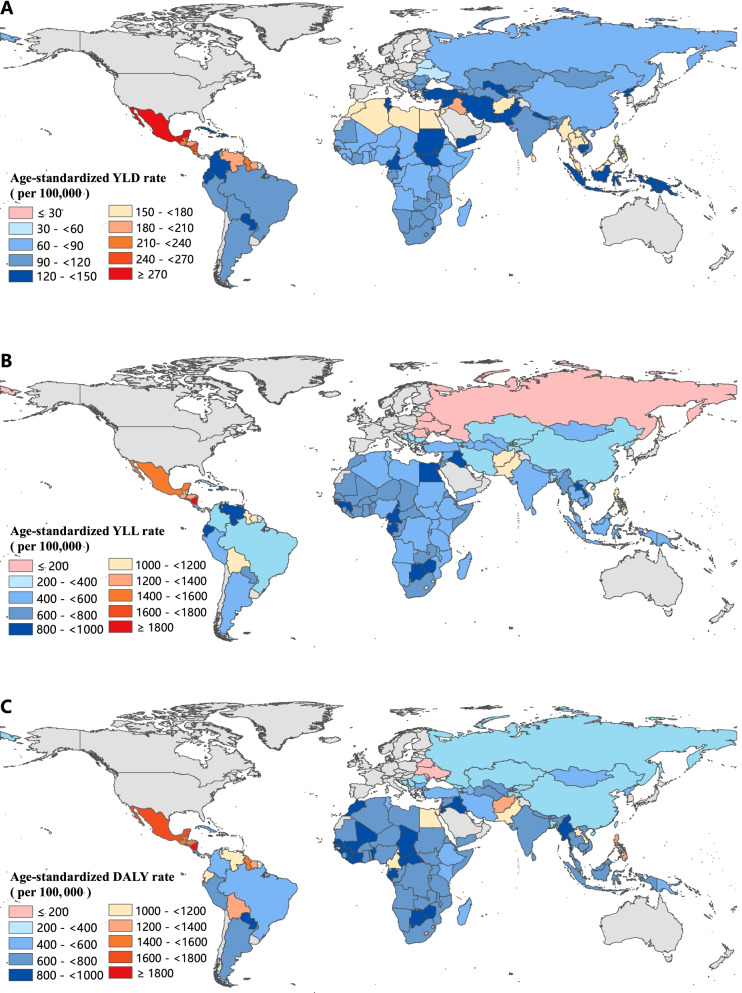
Age-standardized YLD (**A**), YLL (**B**) and DALY (**C**) rates of CKD across 137 low-and middle-income countries, 2019. YLD=Years lived with disability. YLL = Years of life lost. DALY=Disability-adjusted life-years

Among all 137 LMICs, the highest age-standardized YLD, YLL and DALY rates of CKD in 2019 were seen in Mexico 296.40 per 100,000 people (95% UI: 206.99 to 394.46), Micronesia (Federated States of) 1895.00 per 100,000 people (95% UI: 1313.85 to 2478.01) and Micronesia (Federated States of) 2162.73 per 100,000 people (95% UI: 1584.61 to 2761.55), respectively. Whereas the lowest age-standardized YLD, YLL and DALY rates were seen in Ukraine 56.53 per 100,000 people (95% UI: 39.81 to 75.38), Belarus 69.79 per 100,000 people (95% UI: 55.13 to 89.34) and Belarus 128.92 per100 000 people (95% UI: 104.56 to 156.45), respectively. From 1990 to 2019, the growth of age-standardized DALY rate varied from − 46.83% in Mongolia to 182.92% in El Salvador. The variations in other CKD burden metrics (YLDs, YLLs and DALYs) by country in 2019 can be found in Fig. 2 and Additional file.

**Fig. 3:**
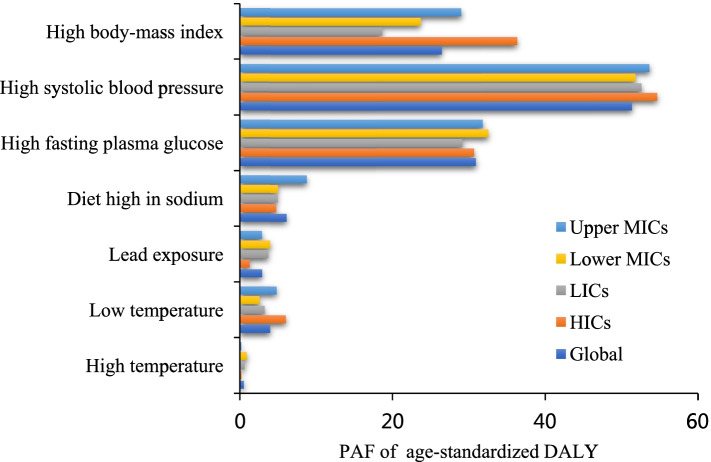
PAF (%) of age-standardized DALY for CKD risk factors, 2019. PAF = Population-attributable faction. DALY=Disability-adjusted life-years. LICs = Low-income countries. Lower MICs = Lower-middle income countries. Upper MICs = Upper-middle income countries. HICs = High-income countries

There was much consistency in the PAF of CKD-related age-standardized DALYs for these risk factors in various income regions. In 2019, CKD-related risk factors with the top three PAF were high systolic blood pressure, high fasting plasma glucose and high body-mass index, which all are metabolic risks (Fig. 3). The PAF of CKD-related age-standardized DALYs for risk factors in 2019 were presented in Table 4.

**Table 4:** PAF (%) of age-standardized DALY for CKD risk factors in specific region, 1990–2019

region	1990PAF	2019PAF	% change	AAPC	*P*-Value
**Global**					
High temperature	−0.07(−4.44 to 2.70)	0.53(−1.52 to 2.05)	−903.97(− 1125.73 to 1395.84)	–	–
Low temperature	4.55(2.60 to 6.43)	3.97(2.15 to 5.60)	−12.71(−25.62 to 1.70)	− 0.6(− 0.7 to − 0.5)	< 0.001
Lead exposure	3.16(1.90 to 4.63)	2.92(1.70 to 4.33)	−7.60(− 13.74 to −3.33)	− 0.2(− 0.4 to − 0.1)	0.003
Diet high in sodium	6.65(2.12 to 13.92)	6.11(1.50 to 13.95)	−8.20(− 34.98 to 1.56)	− 0.2(− 0.3 to − 0.2)	< 0.001
High fasting plasma glucose	29.15(24.92 to 33.18)	30.89(26.49 to 34.95)	5.99(1.48 to 9.25)	0.2(0.2 to 0.3)	< 0.001
High systolic blood pressure	47.48(42.63 to 52.14)	51.36(45.96 to 56.42)	8.17(4.17 to 11.30)	0.3(0.3 to 0.3)	< 0.001
High body-mass index	15.80(8.35 to 25.53)	26.46(16.69 to 37.46)	67.48(42.97 to 105.85)	1.9(1.8 to 1.9)	< 0.001
**HICs**					
High temperature	0.06 (−0.37 to 0.30)	0.22(− 0.36 to 0.59)	248.12(− 1088.38 to 1044.41)	–	–
Low temperature	6.47 (3.83 to 9.26)	5.99(3.53 to 8.53)	−7.33(− 14.91 to 3.27)	− 0.4(− 0.5 to − 0.3)	< 0.001
Lead exposure	1.70 (0.57 to 3.08)	1.28(0.34 to 2.57)	−24.61(−42.43 to − 16.04)	−1.0(− 1.1 to − 0.8)	< 0.001
Diet high in sodium	5.46 (1.22 to 13.14)	4.73(0.57 to 12.79)	− 13.34(− 55.06 to − 1.63)	− 0.7(− 0.7 to − 0.6)	< 0.001
High fasting plasma glucose	27.10 (22.85 to 31.16)	30.67(26.07 to 34.97)	13.14(9.99 to 16.45)	0.4(0.3 to 0.5)	< 0.001
High systolic blood pressure	57.45 (51.66 to 62.62)	54.68(49.03 to 60.1)	−4.82(−6.9 to − 2.81)	− 0.3(− 0.3 to − 0.2)	< 0.001
High body-mass index	27.88 (16.65 to 40.47)	36.3(24.02 to 49.32)	30.23(18.03 to 51.6)	0.9(0.8 to 1.0)	< 0.001
**LICs**					
High temperature	− 0.02(− 7.62 to 4.55)	0.63(− 3.67 to 2.40)	− 3026.12(− 877.52 to 676.46)	–	–
Low temperature	3.71(1.90 to 5.38)	3.24(1.46 to 4.93)	− 12.62(− 26.55 to 5.47)	–0.6(–0.7 to –0.5)	< 0.001
Lead exposure	3.66(2.29 to 5.20)	3.73(2.34 to 5.24)	1.73(− 6.07 to 8.71)	0.0(-0.1 to 0.2)	0.474
Diet high in sodium	5.71(0.86 to 15.00)	4.92(0.48 to 14.20)	−13.83(− 51.75 to − 1.40)	−0.6(−0.6 to −0.6)	< 0.001
High fasting plasma glucose	28.49(23.32 to 33.25)	29.15(24.25 to 33.42)	2.31(− 2.66 to 6.55)	0.1(0.0 to 0.1)	< 0.001
High systolic blood pressure	48.85(43.66 to 54.11)	52.62(47.37 to 57.61)	7.72(0.82 to 11.89)	0.3(0.2 to 0.3)	< 0.001
High body-mass index	10.94(4.62 to 20.08)	18.64(10.40 to 28.84)	70.37(40.14 to 133.32)	2.0(1.8 to 2.2)	< 0.001
**Lower MICs**					
High temperature	−0.14(−8.82 to 5.31)	0.90(− 2.38 to 3.56)	− 729.61(− 1321.80 to 1093.38)	–	–
Low temperature	2.59(0.50 to 4.49)	2.56(0.23 to 4.52)	−1.32(− 50.39 to 36.88)	−0.4(−0.6 to −0.2)	< 0.001
Lead exposure	4.20(2.79 to 5.79)	3.97(2.60 to 5.50)	− 5.35(− 11.02 to − 0.82)	−0.2(−0.4 to −0.1)	0.012
Diet high in sodium	5.15(0.98 to 12.41)	4.97(0.62 to 12.68)	−3.53(− 39.12 to 4.48)	−0.1(−0.1 to 0.0)	< 0.001
High fasting plasma glucose	30.02(25.17 to 34.53)	32.52(27.66 to 37.00)	8.33(3.62 to 12.33)	0.3(0.3 to 0.4)	< 0.001
High systolic blood pressure	48.13(43.14 to 53.29)	51.82(46.08 to 57.17)	7.66(2.87 to 11.00)	0.3(0.3 to 0.3)	< 0.001
High body-mass index	11.18(5.19 to 19.70)	23.68(14.51 to 34.14)	111.83(66.28 to 196.48)	2.7(2.7 to 2.8)	< 0.001
**Upper MICs**					
High temperature	−0.03(−1.47 to 0.82)	0.21(−0.67 to 0.77)	−858.03(− 1391.68 to 1289.47)	–	–
Low temperature	5.83(3.17 to 8.40)	4.79(2.78 to 6.75)	−17.73(−26.50 to −3.66)	− 0.8(−0.9 to −0.7)	< 0.001
Lead exposure	3.07(1.82 to 4.52)	2.90(1.67 to 4.37)	−5.67(− 11.73 to − 0.70)	0.0(-0.1 to 0.2)	0.846
Diet high in sodium	9.49(3.90 to 17.23)	8.74(3.05 to 17.12)	−7.83(− 30.95 to 5.14)	− 0.1(-0.2 to 0.0)	0.041
High fasting plasma glucose	31.37(27.18 to 35.03**)**	31.79(27.68 to 35.63)	1.35(− 2.72 to 5.80)	0.1(0.0 to 0.1)	< 0.001
High systolic blood pressure	45.87(40.97 to 50.79)	53.64(48.10 to 59.04)	16.94(12.55 to 22.01)	0.6(0.6 to 0.7)	< 0.001
High body-mass index	16.71(8.67 to 27.05)	29.01(18.14 to 41.21)	73.58(48.84 to 113.01)	1.9(1.9 to 2.0)	< 0.001

**Fig. 4:**
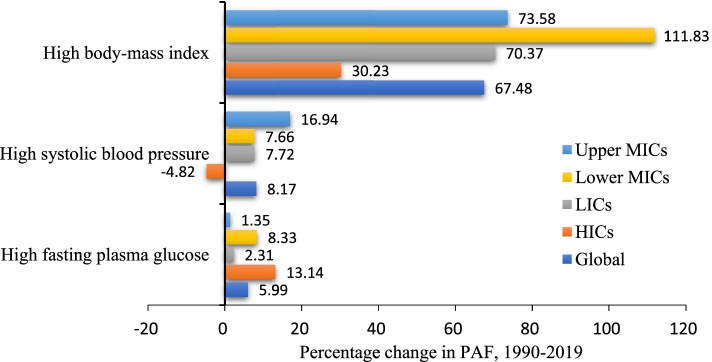
Percentage change (%) in PAF contributions of major risk factors of CKD in specific region, 1990–2019. PAF = Population-attributable faction. LICs = Low-income countries. Lower MICs = Lower-middle income countries. Upper MICs = Upper-middle income countries. HICs = High-income countries

From 1990 to 2019, there were upward trends in the PAF contributions of high fasting plasma glucose, high systolic blood pressure, and high body-mass index to age-standardized CKD DALY in Global, LICs, Lower MICs and Upper MICs (Table 4). Notably, the greatest increase in the PAF was attributed to high body-mass in various income regions, especially in Lower MICs (111.83% (95% UI: 66.28 to 196.48); AAPC = 2.7, 95%UI: 2.7 to 2.8, *P* < 0.001), followed by Upper MICs (73.58% (95% UI: 48.84 to 113.01); AAPC = 1.9, 95%UI: 1.9 to 2.0, *P* < 0.001) and LICs (70.37% (95% UI: 40.14 to 133.32); AAPC = 2.0, 95%UI: 1.8 to 2.2, *P* < 0.001). In addition, the PAF of age-standardized DALYs for high systolic blood pressure increased the most in Upper MICs (16.94% (95% UI: 12.55 to 22.01); AAPC = 0.6, 95%UI: 0.6 to 0.7, *P* < 0.001), whereas decreased in HICs (4.82% (95% UI: − 6.9 to − 2.81); AAPC = -0.3, 95%UI: − 0.3 to − 0.2, *P* < 0.001) (Fig. 4).

## Discussion

Chronic kidney disease (CKD) is a significant global health problem and one of the top 20 causes of death worldwide. It is a serious threat to people’s life and health [26, 27]. CKD DALYs is calculated by adding the YLLs and the YLDs, we found that the age-standardized YLL rate is much higher than the YLD rate at the global level and in various income regions, suggesting that premature death is the main cause of CKD burden. The age-standardized YLD rates all showed an upward trend in LICs, Lower MICs and Upper MICs, the age-standardized YLL rates decreased in LICs and Upper MICs, suggesting that future CKD treatment strategies should focus on improving the quality of life of CKD patients.

Our results show disparities in CKD burden were also observed in geographical regions and countries. LICs had the highest age-standardized DALY rate, followed by Lower MICs, which may be related to different exposures to risk factors, weak health systems, inadequate health care funding, access to effective health care interventions [28]. These findings call for increased planning and interventions, and developed novel and more appropriate solutions to address the burden of CKD in these areas [10]. The greatest decline in age-standardized DALY rates of CKD were all seen in LICs. This may be related to the improvement of medical technology and effective health care reform. However, the age-standardized DALY rate showed an upward trend in Lower MICs. If these trends continue, the disparities that exist in CKD burden between Lower MICs and LICs and Upper MICs will be further increased.

Furthermore, our results showed that the CKD burden showed homogeneity by gender among global and LMICs: age-standardized YLD rate was higher in females than in males, whereas age-standardized rates of YLL and DALY of CKD were all higher in males than in females. The disease burden of CKD is mainly due to premature death in males and disability in females. This difference may be related to physiological differences, estrogen levels and lifestyle between males and females [29]. This gender difference should also be taken into account by policy makers when planning future strategies and implementing preventive and control measures. Additionally, the YLD, YLL and DALY rates of CKD increased with age, which were higher in aged ≥70 years in various income regions, speculated this change is largely related to the aging population and the increase of the elderly population. More attention should be paid to the older adults in the prevention and treatment of CKD.

In 2019, except for age and other non-modifiable risk factors, high systolic blood pressure, high fasting plasma glucose, and high body-mass index remained the major causes attributable age-standardized CKD DALY in LMICs. Unfortunately, there was no remarkable decline in PAF of them since 1990. This may be related to the increased incidence and prevalence of hypertension, dyslipidemia, overweight and obesity and sedentary lifestyle in the population over the past 30 years [30]. Therefore, more efforts are needed to create healthy lifestyles. Moreover, there were upward trends in the PAF of DALYs contributions of high systolic blood pressure, high fasting plasma glucose, and high body-mass in LMICs from 1990 to 2019. Perhaps most concerning are high body mass index, especially in Lower MICs, so it is imperative that effective measures be taken to curb the rise of obesity. There is clear evidence that interventions to manage hypertension and promote weight loss are associated with reduced risks of developing CKD and better outcomes among those living with CKD [31–33]. Therefore, it is important to develop relevant strategies and targeted measures to prevent and manage hypertension, diabetes, and obesity.

GBD 2019 for the first time included high and low temperatures exposure as a global risk factor [13]. This study indicated that the PAF attributable to low temperature exceeded the PAF attributable to high temperature in various income regions in 2019, but the PAF attributable to low temperature showed a downward trend. Generally, infrastructure, such as the spread of heating, housing insulation, and the prevalence of air conditioning are likely explanations [34]. In 1990, high temperatures displayed a protective effect, leading to a negative high-attributable PAF in LMICs. In the context of global climate change, however, the PAF of high temperatures induced CKD is increasing in various income regions, requiring more attention and intervention. Meanwhile, this study shows that LICs and Lower MICs have larger PAF due to high temperature in 2019. This may be because, compared to HICs and Upper MICs, LICs and Lower MICs has higher poverty ratios, fewer medical resources and temperature-control facilities, and most LICs and Lower MICs are in Africa having high exposure to heat [35, 36]. Climate warming may be a potential factor in exacerbating the inter-country health and socio-economic inequalities [35, 37]. Therefore, it is necessary to consider the impact of high temperature and take measures to improve climate resilience, especially in LICs and Lower MICs.

In addition to the seven risk factors for CKD quantified by GBD 2019, there are other potential and nontraditional factors that are likely to affect future global burden of CKD, for example, food insecurity, heavy metals, air pollution, persistent organic pollutants, war and displaced refugee populations and so on [38–41]. Further study of potential risk factors for CKD is also necessary in order to facilitate the development of targeted interventions. In addition, in the context of the pandemic of coronavirus disease 2019, although COVID-19 predominantly affects the respiratory system, it also involves multiple organs, such as the cardiovascular system, the liver as well as the kidneys [42]. Adverse renal manifestations, such as acute kidney injury (AKI), have been reported as a result of COVID-19. Studies have shown that AKI can directly cause ESRD, and increase the risk of developing incident CKD and worsening of underlying CKD [8]. CKD has not been sufficiently recognized due to its inconspicuous course during COVID-19 infection, especially in the early stage [42]. As the pandemic continues, more precise prevention policies may be needed to guide patients with chronic kidney disease. COVID-19 is a huge medical challenge that health care providers should recognize early and take appropriate action as soon as possible.

### Limitations

Our study has several limitations. First, since data for this study is part of GBD 2019 study, all limitations of the GBD 2019 methods outlined elsewhere also apply here [9, 13]. The major limitation of the GBD analysis of the burden of diseases is the availability of primary data [9]. For exposure measurement, patterns of data availability are non-uniform across geography and over time and, where available, might be based on less reliable modes of data collection such as self-report [13]. Then, In the estimation of YLDs, the disability weight is derived from statistical data of multiple countries around the world, which is uncertainty in the estimation of disease in LMICs. In addition, a comprehensive assessment of the burden of disease should also include the economic, family and social aspects of the burden, so a multi-dimensional analysis can be considered to improve the accuracy of the results [43].

## Conclusions

This study has evaluated the burden of CKD and its risk-attributable burden in 137 LMICs from 1990 to 2019. CKD burden remains high in various income regions, especially in LICs and Lower MICs. More effective and targeted preventive policies and interventions aimed at mitigating preventable CKD burden and addressing risk factors are urgently needed, particularly in geographies with high or increasing burden.

## Supplementary Information

**Additional file 1: Table S1**. Number of YLDs, YLLs, DALYs of CKD with percent change in 137 low-and middle-income countries, 1990–2019. **Table S2**. Age-standardized YLD, YLL and DALY rates of CKD with percent change in 137 low-and middle-income countries, 1990–2019. **Table S3**. The AAPC in age-standardized rate of YLD, YLL, and DALY for CKD in 137 low-and middle-income countries, 1990–2019.

## Data Availability

Data for this study were obtained from the GBD 2019 study. This study adherence to the Guidelines for Accurate and Transparent Health Estimates Reporting (GATHER) recommendations. All data used in this study were obtained from the Institute for Health Metrics and Evaluation (IHME) website (http://ghdx.healthdata.org/gbd-results-tool) [20]. And the public access to the database is open.

## Abbreviations

GBD: Global Burden of Disease
CKD: Chronic kidney disease
LMICs: Low - and middle-income countries
LICs: Low-income countries
Lower MICs: Lower-middle income countries
Upper MICs: Upper-middle income countries
HICs: High-income countries
YLD: Years lived with disability
YLL: Years of life lost
DALY: Disability-adjusted life-years
PAF: Population-attributable faction
UI: Uncertainty interval
AAPC: Average annual percentage change
AKI: Acute kidney injury
ESRD: End-stage renal disease
COVID-19: Coronavirus disease 2019
