# Correction to: Burden of chronic kidney disease and its risk-attributable burden in 137 low-and middle-income countries, 1990–2019: results from the global burden of disease study 2019

**DOI:** 10.1186/s12882-022-02686-x

**Published:** 2022-02-14

**Authors:** Changrong Ke, Juanjuan Liang, Mi Liu, Shiwei Liu, Chunping Wang

**Affiliations:** 1 School of Public Health, Weifang Medical University, Weifang, 261053 China; 2 Chinese Center for Disease Control and Prevention, Beijing, 102206 China

**Correction to: BMC Nephrol 23, 17 (2022)**

**https://doi.org/10.1186/s12882-021-02597-3**

Following publication of the original article [1], the authors identified an error in Fig. 2. The correct figure is given below.

**Fig. 2:**
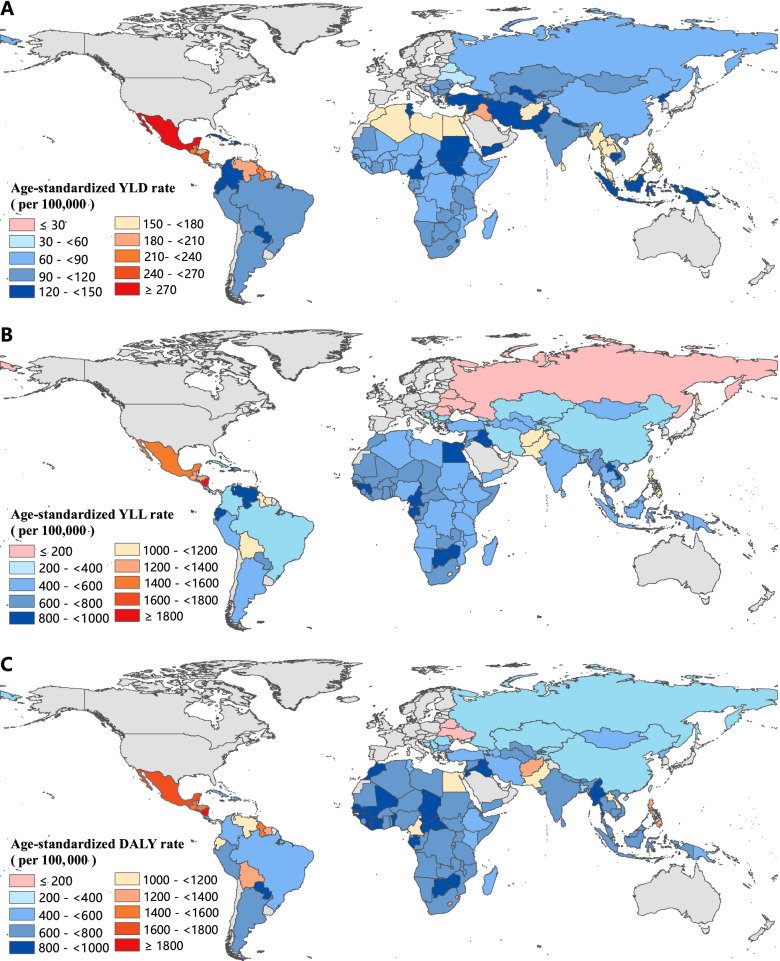
Age-standardized YLD (**A**), YLL (**B**) and DALY (**C**) rates of CKD across 137 low-and middle-income countries, 2019. YLD=Years lived with disability. YLL = Years of life lost. DALY=Disability-adjusted life-years

The original article has been corrected.
