# Supplementary material for: Burden of chronic kidney disease and its risk-attributable burden in 137 low-and middle-income countries, 1990–2019: results from the global burden of disease study 2019
Source: BMC Nephrol. 2022 Jan 5;23:17. doi: 10.1186/s12882-021-02597-3 (PMC8727977; doi:10.1186/s12882-021-02597-3)
Supplement: Supplementary file 1 — Additional file 1: Table S1. Number of YLDs, YLLs, DALYs of CKD with percent change in 137 low-and middle-income countries, 1990–2019. Table S2. Age-standardized YLD, YLL and DALY rates of CKD with percent change in 137 low-and middle-income countries, 1990–2019. Table S3. The AAPC in age-standardized rate of YLD, YLL, and DALY for CKD in 137 low-and middle-income countries, 1990–2019. [file 12882_2021_2597_MOESM1_ESM.docx]

Table S1. Number of YLDs, YLLs, DALYs of CKD with percent change in 137 low-and middle-income countries, 1990 - 2019

| country | **YLDs (in thousands)** | | | **YLLs (in thousands)** | | | **DALYs (in thousands)** | | |
| --- | --- | --- | --- | --- | --- | --- | --- | --- | --- |
|  | 1990 | 2019 | % change | 1990 | 2019 | % change | 1990 | 2019 | % change |
| **LICs** |  |  |  |  |  |  |  |  |  |
| Afghanistan | 8.28(6.04 to 10.90) | 32.52(23.34 to 43.08) | 292.68 | 112.84(89.41 to 145.79) | 187.01(143.74 to 257.71) | 65.73 | 121.12(97.36 to 154.91) | 219.52(174.84 to 290.79) | 81.24 |
| Benin | 2.10(1.48 to 2.83) | 7.25(5.20 to 9.73) | 246.19 | 25.59(21.29 to 30.48) | 54.03(38.43 to 73.35) | 111.10 | 27.69(23.32 to 32.63) | 61.28(45.03 to 81.49) | 121.33 |
| Burkina Faso | 3.72(2.63 to 4.96) | 12.66(9.06 to 16.81) | 240.54 | 43.73(35.12 to 52.78) | 103.49(80.18 to 131.21) | 136.64 | 47.45(38.64 to 56.35) | 116.15(91.48 to 143.87) | 144.78 |
| Burundi | 2.11(1.49 to 2.83) | 5.02(3.52 to 6.70) | 138.07 | 25.18(19.02 to 32.09) | 34.67(26.01 to 44.80) | 37.68 | 27.29(21.09 to 34.35) | 39.69(30.86 to 50.19) | 45.44 |
| Central African Republic | 0.94(0.66 to 1.26) | 2.34(1.58 to 3.14) | 148.54 | 13.51(10.25 to 17.07) | 24.19(17.70 to 32.71) | 79.05 | 14.45(11.13 to 18.06) | 26.53(19.95 to 35.04) | 83.58 |
| Chad | 2.46(1.78 to 3.26) | 7.95(5.59 to 10.74) | 223.44 | 30.38(23.34 to 42.13) | 65.04(47.54 to 87.92) | 114.12 | 32.83(25.93 to 44.61) | 72.99(55.43 to 96.04) | 122.31 |
| Democratic People's Republic of Korea | 20.87(15.01 to 27.59) | 41.00(29.72 to 54.58) | 96.42 | 77.89(58.54 to 100.20) | 112.06(88.28 to 141.61) | 43.87 | 98.77(78.30 to 121.95) | 153.07(124.66 to 184.16) | 54.98 |
| Democratic Republic of the Congo | 13.71(9.78 to 18.31) | 38.09(26.92 to 50.89) | 177.82 | 165.57(134.52 to 200.29) | 261.33(204.03 to 326.00) | 57.84 | 179.28(147.45 to 213.53) | 299.42(238.30 to 369.82) | 67.01 |
| Eritrea | 1.05(0.74 to 1.40) | 3.47(2.39 to 4.61) | 230.98 | 9.85(6.86 to 13.21) | 22.69(15.07 to 32.92) | 130.24 | 10.90(7.90 to 14.20) | 26.16(18.40 to 36.10) | 139.93 |
| Ethiopia | 19.46(13.63 to 25.84) | 48.95(34.76 to 65.27) | 151.54 | 295.75(225.52 to 359.45) | 280.75(244.68 to 324.65) | -5.07 | 315.21(244.41 to 377.36) | 329.70(289.16 to 376.16) | 4.60 |
| Gambia | 0.41(0.29 to 0.55) | 1.34(0.95 to 1.79) | 224.43 | 3.78(2.70 to 5.09) | 9.09(6.80 to 11.93) | 140.25 | 4.20(3.10 to 5.53) | 10.43(8.04 to 13.31) | 148.52 |
| Guinea | 2.95(2.11 to 3.93) | 7.44(5.30 to 10.00) | 151.89 | 42.42(34.01 to 54.79) | 63.68(47.81 to 83.07) | 50.12 | 45.37(36.83 to 57.55) | 71.12(55.25 to 90.87) | 56.74 |
| Guinea-Bissau | 0.50(0.35 to 0.67) | 1.23(0.87 to 1.65) | 146.69 | 7.99(6.05 to 10.20) | 10.44(8.06 to 13.31) | 30.68 | 8.49(6.61 to 10.69) | 11.67(9.26 to 14.67) | 37.51 |
| Haiti | 5.44(3.89 to 7.19) | 15.97(11.48 to 21.16) | 193.72 | 43.28(33.44 to 57.71) | 76.85(54.35 to 112.76) | 77.55 | 48.72(38.93 to 62.48) | 92.82(69.30 to 130.33) | 90.51 |
| Liberia | 0.97(0.69 to 1.29) | 2.80(1.96 to 3.74) | 189.19 | 14.96(11.85 to 18.32) | 20.62(14.41 to 29.30) | 37.86 | 15.92(12.74 to 19.33) | 23.42(17.07 to 32.08) | 47.07 |
| Madagascar | 4.60(3.28 to 6.18) | 12.30(8.63 to 16.53) | 167.27 | 43.22(32.43 to 53.51) | 64.54(49.53 to 84.75) | 49.34 | 47.82(37.19 to 58.03) | 76.84(61.02 to 96.80) | 60.69 |
| Malawi | 3.82(2.70 to 5.08) | 9.40(6.58 to 12.51) | 146.39 | 39.04(26.10 to 47.48) | 56.04(44.59 to 68.94) | 43.52 | 42.86(29.95 to 51.31) | 65.44(53.60 to 79.12) | 52.68 |
| Mali | 4.00(2.87 to 5.28) | 12.22(8.62 to 16.43) | 205.85 | 56.02(41.90 to 71.67) | 95.24(68.99 to 128.09) | 70.01 | 60.02(45.84 to 75.74) | 107.46(81.26 to 140.09) | 79.05 |
| Mozambique | 4.86(3.47 to 6.38) | 14.83(10.59 to 19.65) | 205.03 | 43.67(35.10 to 53.14) | 90.82(68.51 to 116.40) | 107.95 | 48.54(39.68 to 58.40) | 105.65(82.50 to 130.23) | 117.68 |
| Nepal | 9.62(6.74 to 12.68) | 30.84(22.12 to 40.55) | 220.45 | 60.85(45.09 to 77.90) | 138.06(95.64 to 184.88) | 126.89 | 70.47(54.03 to 88.02) | 168.90(126.05 to 216.92) | 139.66 |
| Niger | 3.96(2.80 to 5.27) | 12.90(9.22 to 17.07) | 225.75 | 45.97(34.50 to 59.04) | 82.20(58.01 to 112.74) | 78.82 | 49.93(38.21 to 62.79) | 95.10(70.71 to 126.27) | 90.48 |
| Rwanda | 2.80(1.96 to 3.76) | 6.50(4.50 to 8.74) | 131.78 | 32.63(25.48 to 39.31) | 37.66(28.53 to 47.84) | 15.42 | 35.43(28.28 to 42.19) | 44.16(35.14 to 54.38) | 24.63 |
| Sierra Leone | 1.70(1.22 to 2.27) | 4.94(3.49 to 6.61) | 190.55 | 21.52(16.04 to 27.78) | 35.68(25.81 to 48.09) | 65.76 | 23.23(17.56 to 29.40) | 40.62(30.39 to 53.27) | 74.90 |
| Somalia | 2.83(1.99 to 3.78) | 9.38(6.59 to 12.50) | 231.15 | 28.44(21.22 to 36.21) | 70.42(53.34 to 93.45) | 147.56 | 31.28(23.91 to 39.04) | 79.80(61.96 to 102.46) | 155.14 |
| South Sudan | 2.14(1.51 to 2.86) | 4.25(2.98 to 5.67) | 98.17 | 21.72(15.92 to 28.41) | 29.47(21.61 to 40.23) | 35.69 | 23.86(18.18 to 30.67) | 33.72(25.30 to 44.73) | 41.30 |
| Syrian Arab Republic | 9.56(6.86 to 12.70) | 18.81(13.47 to 24.88) | 96.70 | 64.25(52.91 to 76.14) | 64.74(49.86 to 84.74) | 0.76 | 73.81(62.62 to 86.07) | 83.54(66.91 to 103.28) | 13.19 |
| Tajikistan | 2.34(1.64 to 3.12) | 6.22(4.36 to 8.26) | 166.28 | 3.72(3.43 to 4.09) | 14.00(11.31 to 17.45) | 276.63 | 6.05(5.32 to 6.87) | 20.22(16.86 to 23.91) | 234.03 |
| Togo | 1.61(1.13 to 2.17) | 4.88(3.40 to 6.46) | 202.24 | 15.92(12.49 to 20.46) | 33.11(24.87 to 42.40) | 108.00 | 17.53(14.11 to 22.08) | 37.99(29.48 to 47.68) | 116.68 |
| Uganda | 5.44(3.91 to 7.36) | 16.16(11.42 to 21.54) | 196.85 | 49.64(37.64 to 63.73) | 108.59(81.00 to 141.33) | 118.73 | 55.09(42.83 to 68.90) | 124.75(95.51 to 156.79) | 126.45 |
| United Republic of Tanzania | 10.76(7.61 to 14.20) | 33.23(23.71 to 44.40) | 208.91 | 93.20(75.84 to 110.83) | 188.34(153.10 to 230.18) | 102.08 | 103.96(86.51 to 121.74) | 221.56(183.03 to 266.29) | 113.13 |
| Yemen | 6.66(4.77 to 8.87) | 27.05(19.06 to 35.75) | 306.20 | 39.07(28.00 to 53.71) | 75.19(56.05 to 100.20) | 92.47 | 45.72(34.30 to 60.81) | 102.24(80.53 to 129.33) | 123.60 |
| **Lower MICs** |  |  |  |  |  |  |  |  |  |
| Angola | 3.15(2.19 to 4.22) | 12.80(8.84 to 17.33) | 305.92 | 41.89(29.73 to 54.36) | 85.25(58.91 to 110.33) | 103.54 | 45.04(33.02 to 57.63) | 98.06(71.21 to 122.73) | 117.71 |
| Bangladesh | 36.14(25.71 to 48.02) | 115.18(83.18 to 151.56) | 218.70 | 353.32(188.54 to 481.94) | 359.81(291.69 to 436.16) | 1.84 | 389.46(223.31 to 518.63) | 474.99(396.53 to 561.30) | 21.96 |
| Bhutan | 0.31(0.22 to 0.41) | 0.77(0.56 to 1.02) | 149.15 | 2.01(1.25 to 2.86) | 3.81(2.62 to 5.05) | 89.12 | 2.33(1.54 to 3.15) | 4.58(3.41 to 5.83) | 97.14 |
| Bolivia (Plurinational State of) | 3.64(2.62 to 4.80) | 11.40(8.13 to 15.12) | 212.68 | 38.94(32.35 to 47.92) | 101.21(76.60 to 130.13) | 159.95 | 42.58(36.17 to 51.30) | 112.61(86.97 to 141.15) | 164.46 |
| Cabo Verde | 0.16(0.11 to 0.21) | 0.42(0.29 to 0.55) | 167.51 | 0.99(0.89 to 1.12) | 2.27(1.94 to 2.65) | 129.89 | 1.14(1.03 to 1.28) | 2.69(2.32 to 3.07) | 135.00 |
| Cambodia | 6.12(4.32 to 8.01) | 17.64(12.48 to 23.33) | 188.43 | 46.67(38.44 to 55.65) | 65.72(52.66 to 80.12) | 40.82 | 52.79(44.49 to 61.78) | 83.37(69.49 to 99.09) | 57.92 |
| Cameroon | 5.92(4.13 to 7.84) | 24.37(17.16 to 32.72) | 311.58 | 64.11(49.09 to 79.05) | 160.72(113.49 to 217.40) | 150.71 | 70.03(55.24 to 85.36) | 185.09(136.24 to 243.22) | 164.31 |
| Comoros | 0.22(0.16 to 0.29) | 0.50(0.36 to 0.67) | 129.44 | 1.75(0.80 to 2.35) | 2.89(2.16 to 3.61) | 65.14 | 1.97(1.01 to 2.58) | 3.39(2.64 to 4.15) | 72.32 |
| Congo | 1.00(0.71 to 1.33) | 3.07(2.15 to 4.10) | 206.20 | 12.44(8.71 to 15.85) | 20.98(14.06 to 28.51) | 68.64 | 13.44(9.76 to 16.88) | 24.04(16.94 to 31.75) | 78.89 |
| Côte d'Ivoire | 6.16(4.32 to 8.22) | 18.11(12.74 to 24.03) | 193.93 | 61.00(48.03 to 74.50) | 117.58(83.30 to 153.38) | 92.76 | 67.16(54.22 to 80.68) | 135.69(101.01 to 173.23) | 102.04 |
| Djibouti | 0.18(0.12 to 0.24) | 0.75(0.52 to 1.01) | 323.38 | 1.20(0.86 to 1.61) | 4.21(2.92 to 6.03) | 250.62 | 1.38(1.03 to 1.78) | 4.97(3.67 to 6.66) | 260.00 |
| Egypt | 40.09(28.97 to 53.12) | 127.09(91.67 to 168.12) | 217.04 | 274.73(191.87 to 335.50) | 529.18(304.74 to 766.08) | 92.62 | 314.81(230.31 to 377.51) | 656.27(428.03 to 899.78) | 108.46 |
| El Salvador | 5.09(3.58 to 6.81) | 14.74(10.23 to 19.57) | 189.59 | 19.17(18.08 to 20.41) | 99.00(74.28 to 130.77) | 416.51 | 24.26(22.30 to 26.46) | 113.74(88.60 to 146.55) | 368.90 |
| Eswatini | 0.45(0.31 to 0.61) | 1.09(0.77 to 1.47) | 140.15 | 3.22(2.53 to 3.94) | 8.26(5.67 to 11.08) | 156.24 | 3.68(2.95 to 4.42) | 9.35(6.62 to 12.20) | 154.25 |
| Ghana | 6.21(4.42 to 8.45) | 21.07(15.05 to 27.97) | 239.54 | 57.55(42.67 to 76.21) | 144.81(102.28 to 187.46) | 151.63 | 63.76(48.45 to 83.00) | 165.88(123.30 to 208.55) | 160.19 |
| Honduras | 3.81(2.69 to 5.06) | 13.81(9.84 to 18.40) | 261.89 | 18.68(15.45 to 23.11) | 78.74(62.19 to 103.38) | 321.61 | 22.49(19.09 to 27.17) | 92.55(75.05 to 118.45) | 311.48 |
| India | 615.73(435.58 to 800.43) | 1328.75(949.75 to 1735.99) | 115.80 | 3063.69(2632.63 to 3474.41) | 6190.94(5333.66 to 7155.21) | 102.07 | 3679.42(3212.89 to 4130.78) | 7519.69(6550.35 to 8500.58) | 104.37 |
| Indonesia | 137.70(97.13 to 183.65) | 341.90(241.48 to 455.04) | 148.29 | 870.37(767.52 to 968.65) | 1297.27(1106.79 to 1527.69) | 49.05 | 1008.07(899.29 to 1118.40) | 1639.17(1421.35 to 1894.19) | 62.61 |
| Kenya | 7.44(5.23 to 9.97) | 24.07(16.93 to 32.34) | 223.74 | 49.80(42.26 to 58.95) | 138.71(113.66 to 167.24) | 178.56 | 57.23(49.08 to 66.49) | 162.79(137.62 to 191.12) | 184.43 |
| Kiribati | 0.09(0.06 to 0.12) | 0.22(0.15 to 0.28) | 138.88 | 0.59(0.48 to 0.71) | 1.27(0.94 to 1.66) | 115.27 | 0.68(0.57 to 0.81) | 1.49(1.13 to 1.89) | 118.39 |
| Kyrgyzstan | 3.24(2.27 to 4.34) | 5.71(4.01 to 7.67) | 76.05 | 17.68(15.96 to 19.46) | 17.58(15.18 to 20.19) | -0.55 | 20.92(18.91 to 22.86) | 23.29(20.32 to 26.54) | 11.31 |
| Lao People's Democratic Republic | 3.34(2.38 to 4.37) | 9.47(6.75 to 12.63) | 183.72 | 37.61(29.37 to 47.17) | 50.84(37.41 to 65.67) | 35.19 | 40.94(32.74 to 50.30) | 60.31(46.61 to 75.59) | 47.30 |
| Lesotho | 0.92(0.65 to 1.23) | 1.77(1.24 to 2.41) | 93.64 | 6.20(4.94 to 7.75) | 16.29(11.25 to 21.93) | 162.83 | 7.11(5.81 to 8.65) | 18.06(12.78 to 23.91) | 153.92 |
| Mauritania | 1.09(0.77 to 1.46) | 2.83(2.00 to 3.74) | 159.41 | 13.52(11.21 to 15.96) | 15.43(10.61 to 20.94) | 14.12 | 14.61(12.31 to 16.99) | 18.25(13.42 to 23.97) | 24.95 |
| Micronesia (Federated States of) | 0.12(0.08 to 0.15) | 0.23(0.17 to 0.31) | 99.74 | 0.74(0.58 to 0.94) | 1.53(1.02 to 2.05) | 106.18 | 0.86(0.69 to 1.07) | 1.76(1.25 to 2.29) | 105.30 |
| Mongolia | 1.48(1.05 to 1.97) | 3.33(2.35 to 4.43) | 125.39 | 13.78(11.80 to 16.09) | 13.31(10.31 to 17.35) | -3.41 | 15.26(13.15 to 17.61) | 16.65(13.43 to 20.69) | 9.08 |
| Morocco | 15.69(11.23 to 20.58) | 53.45(38.30 to 70.75) | 240.74 | 97.30(83.95 to 114.19) | 206.87(157.88 to 260.15) | 112.61 | 112.99(98.97 to 130.44) | 260.33(206.44 to 316.87) | 130.40 |
| Myanmar | 38.35(27.14 to 50.64) | 88.70(63.63 to 117.15) | 131.30 | 298.45(224.86 to 377.08) | 348.68(275.82 to 438.29) | 16.83 | 336.80(265.44 to 413.27) | 437.37(358.74 to 530.90) | 29.86 |
| Nicaragua | 3.63(2.54 to 4.84) | 13.67(9.30 to 18.66) | 276.90 | 20.03(18.67 to 21.63) | 88.94(71.89 to 107.43) | 344.06 | 23.65(21.83 to 25.60) | 102.61(84.70 to 122.82) | 333.77 |
| Nigeria | 40.51(28.54 to 53.78) | 122.79(86.96 to 163.81) | 203.12 | 390.06(309.74 to 502.57) | 645.01(488.28 to 832.62) | 65.36 | 430.56(348.85 to 544.61) | 767.80(610.72 to 961.31) | 78.32 |
| Pakistan | 67.43(47.74 to 88.55) | 194.44(137.88 to 255.41) | 188.36 | 481.68(351.24 to 649.56) | 1523.82(1105.56 to 1950.80) | 216.36 | 549.11(415.15 to 718.95) | 1718.27(1288.11 to 2139.72) | 212.92 |
| Palestine | 1.56(1.12 to 2.06) | 5.46(3.92 to 7.21) | 249.70 | 10.66(8.49 to 13.16) | 16.39(14.11 to 18.80) | 53.72 | 12.22(9.91 to 14.77) | 21.85(19.03 to 24.72) | 78.76 |
| Papua New Guinea | 2.60(1.83 to 3.46) | 9.11(6.32 to 12.20) | 250.81 | 10.42(8.13 to 12.70) | 29.22(22.06 to 37.89) | 180.46 | 13.01(10.65 to 15.42) | 38.33(30.61 to 47.77) | 194.49 |
| Philippines | 47.33(33.51 to 62.52) | 161.44(112.44 to 214.21) | 241.08 | 335.72(307.67 to 365.60) | 987.75(820.48 to 1174.71) | 194.22 | 383.05(352.62 to 418.20) | 1149.19(976.76 to 1336.95) | 200.01 |
| Republic of Moldova | 1.94(1.39 to 2.60) | 2.95(2.10 to 3.90) | 52.01 | 2.52(2.37 to 2.67) | 5.67(4.90 to 6.47) | 124.92 | 4.46(3.88 to 5.12) | 8.62(7.48 to 9.88) | 93.22 |
| Sao Tome and Principe | 0.07(0.05 to 0.10) | 0.18(0.12 to 0.24) | 137.05 | 0.84(0.65 to 0.99) | 1.42(1.12 to 1.76) | 69.98 | 0.91(0.72 to 1.07) | 1.60(1.29 to 1.95) | 75.46 |
| Senegal | 3.52(2.46 to 4.68) | 8.63(6.12 to 11.40) | 145.30 | 45.42(34.07 to 59.89) | 71.36(51.27 to 96.99) | 57.10 | 48.94(37.65 to 63.02) | 79.99(59.36 to 105.45) | 63.44 |
| Solomon Islands | 0.38(0.27 to 0.51) | 1.03(0.73 to 1.36) | 168.23 | 2.66(1.98 to 3.57) | 3.54(2.77 to 4.37) | 33.05 | 3.04(2.36 to 3.94) | 4.56(3.71 to 5.50) | 50.10 |
| Sudan | 10.67(7.71 to 14.12) | 36.11(25.93 to 48.11) | 238.41 | 86.01(62.68 to 113.64) | 129.72(89.66 to 190.55) | 50.82 | 96.68(73.53 to 124.82) | 165.84(122.30 to 227.64) | 71.53 |
| Timor-Leste | 0.46(0.32 to 0.60) | 1.28(0.93 to 1.67) | 181.73 | 3.51(2.60 to 4.48) | 6.25(4.21 to 8.15) | 78.17 | 3.96(3.09 to 4.96) | 7.53(5.44 to 9.52) | 90.08 |
| Tunisia | 5.79(4.13 to 7.77) | 17.35(12.56 to 22.91) | 199.80 | 27.18(23.54 to 31.37) | 51.46(36.75 to 69.07) | 89.29 | 32.97(29.03 to 37.55) | 68.81(53.34 to 87.35) | 108.69 |
| Ukraine | 25.77(18.25 to 34.15) | 32.64(23.36 to 43.55) | 26.62 | 27.40(26.25 to 28.53) | 60.90(51.27 to 71.41) | 122.30 | 53.17(45.22 to 61.53) | 93.54(79.63 to 109.52) | 75.92 |
| Uzbekistan | 15.88(11.21 to 20.95) | 38.31(26.89 to 50.63) | 141.18 | 70.75(62.72 to 88.01) | 148.40(124.45 to 175.27) | 109.76 | 86.63(76.87 to 105.00) | 186.71(158.69 to 216.81) | 115.52 |
| Vanuatu | 0.12(0.09 to 0.17) | 0.41(0.29 to 0.54) | 228.30 | 0.53(0.36 to 0.73) | 2.15(1.54 to 2.91) | 309.10 | 0.65(0.47 to 0.86) | 2.56(1.91 to 3.35) | 293.71 |
| Viet Nam | 42.14(29.89 to 56.15) | 115.01(81.86 to 153.17) | 172.92 | 289.60(231.38 to 359.23) | 494.68(382.14 to 615.19) | 70.81 | 331.74(272.64 to 403.02) | 609.69(487.51 to 737.18) | 83.78 |
| Zambia | 3.55(2.50 to 4.78) | 11.50(8.15 to 15.33) | 224.36 | 35.88(29.79 to 42.18) | 64.53(50.40 to 81.14) | 79.84 | 39.43(33.09 to 45.90) | 76.03(61.01 to 93.36) | 92.84 |
| Zimbabwe | 4.74(3.35 to 6.29) | 10.29(7.26 to 13.60) | 117.11 | 26.23(19.65 to 39.62) | 67.79(48.23 to 99.57) | 158.40 | 30.97(24.10 to 44.64) | 78.08(57.85 to 111.14) | 152.08 |
| **Upper MICs** |  |  |  |  |  |  |  |  |  |
| Albania | 1.66(1.19 to 2.20) | 2.74(1.97 to 3.65) | 64.84 | 7.80(7.25 to 8.44) | 7.13(5.30 to 9.30) | -8.63 | 9.46(8.74 to 10.25) | 9.87(7.82 to 12.19) | 4.26 |
| Algeria | 17.14(12.41 to 22.73) | 57.77(41.84 to 76.66) | 237.10 | 103.47(79.84 to 144.82) | 176.95(137.86 to 236.46) | 71.01 | 120.61(95.86 to 163.58) | 234.72(189.60 to 298.02) | 94.61 |
| American Samoa | 0.05(0.04 to 0.07) | 0.13(0.09 to 0.17) | 131.98 | 0.25(0.21 to 0.29) | 0.66(0.55 to 0.80) | 168.68 | 0.30(0.26 to 0.35) | 0.79(0.67 to 0.93) | 161.99 |
| Argentina | 27.97(20.26 to 36.65) | 48.28(35.42 to 63.06) | 72.65 | 153.73(146.74 to 160.99) | 271.95(252.39 to 293.79) | 76.91 | 181.69(171.13 to 192.95) | 320.23(295.82 to 345.80) | 76.25 |
| Armenia | 1.80(1.25 to 2.40) | 3.18(2.26 to 4.29) | 77.00 | 1.51(1.40 to 1.63) | 8.94(7.50 to 10.49) | 490.63 | 3.31(2.74 to 3.93) | 12.12(10.35 to 13.91) | 266.18 |
| Azerbaijan | 4.72(3.33 to 6.22) | 11.55(8.11 to 15.23) | 144.68 | 19.87(17.52 to 22.36) | 41.18(34.61 to 48.48) | 107.27 | 24.58(21.73 to 27.53) | 52.72(45.07 to 61.52) | 114.45 |
| Belarus | 5.37(3.83 to 7.18) | 7.32(5.24 to 9.82) | 36.22 | 5.11(4.83 to 5.37) | 9.85(7.80 to 12.61) | 92.72 | 10.48(8.90 to 12.32) | 17.17(14.06 to 20.78) | 63.76 |
| Belize | 0.17(0.12 to 0.22) | 0.75(0.53 to 0.99) | 345.19 | 0.63(0.57 to 0.69) | 3.21(2.77 to 3.73) | 407.22 | 0.80(0.72 to 0.88) | 3.96(3.44 to 4.55) | 394.22 |
| Bosnia and Herzegovina | 2.87(2.08 to 3.76) | 4.31(3.09 to 5.79) | 49.99 | 8.93(8.40 to 9.50) | 12.17(9.58 to 15.22) | 36.20 | 11.81(10.79 to 12.88) | 16.47(13.69 to 19.82) | 39.55 |
| Botswana | 0.64(0.45 to 0.86) | 2.16(1.53 to 2.94) | 238.12 | 4.16(2.93 to 5.82) | 13.07(8.68 to 18.32) | 214.43 | 4.79(3.53 to 6.40) | 15.23(10.76 to 20.54) | 217.59 |
| Brazil | 111.67(79.09 to 146.95) | 266.51(192.50 to 350.97) | 138.66 | 489.53(473.32 to 505.00) | 917.60(869.76 to 959.41) | 87.44 | 601.20(565.00 to 639.03) | 1184.12(1093.95 to 1274.56) | 96.96 |
| Bulgaria | 6.79(4.82 to 9.19) | 10.03(7.09 to 13.40) | 47.75 | 19.38(18.31 to 20.44) | 40.54(32.34 to 50.69) | 109.17 | 26.17(23.83 to 28.64) | 50.57(41.68 to 61.76) | 93.23 |
| China | 800.31(574.86 to 1062.72) | 1626.00(1160.36 to 2192.22) | 103.17 | 3196.71(2793.86 to 3595.63) | 4205.85(3571.00 to 4848.08) | 31.57 | 3997.03(3531.28 to 4458.76) | 5831.84(4992.21 to 6645.33) | 45.90 |
| Colombia | 27.66(19.27 to 36.79) | 76.12(53.13 to 103.15) | 175.23 | 104.41(99.89 to 109.01) | 185.15(143.33 to 237.19) | 77.34 | 132.06(122.33 to 142.01) | 261.27(212.26 to 320.89) | 97.84 |
| Costa Rica | 4.07(2.86 to 5.44) | 10.89(7.56 to 14.58) | 167.33 | 6.19(5.77 to 6.62) | 28.53(22.36 to 35.88) | 360.80 | 10.26(9.02 to 11.61) | 39.41(31.70 to 48.27) | 284.04 |
| Cuba | 9.91(7.04 to 13.22) | 21.26(15.21 to 28.76) | 114.50 | 22.44(21.43 to 23.50) | 58.02(46.99 to 71.08) | 158.57 | 32.35(29.35 to 35.80) | 79.28(66.07 to 94.26) | 145.07 |
| Dominica | 0.11(0.08 to 0.15) | 0.18(0.13 to 0.24) | 61.84 | 0.58(0.51 to 0.64) | 0.94(0.76 to 1.15) | 62.68 | 0.69(0.61 to 0.76) | 1.11(0.92 to 1.35) | 62.55 |
| Dominican Republic | 4.83(3.41 to 6.40) | 15.03(10.74 to 19.99) | 211.41 | 20.40(18.24 to 22.80) | 60.54(43.54 to 82.27) | 196.74 | 25.23(22.41 to 28.03) | 75.57(58.12 to 97.46) | 199.54 |
| Ecuador | 5.89(4.12 to 7.95) | 22.95(16.42 to 30.95) | 289.59 | 32.06(30.13 to 34.08) | 146.92(115.98 to 189.93) | 358.26 | 37.95(35.33 to 40.92) | 169.86(137.38 to 212.39) | 347.60 |
| Equatorial Guinea | 0.14(0.10 to 0.19) | 0.71(0.50 to 0.96) | 403.90 | 2.00(1.46 to 2.55) | 4.21(2.82 to 6.26) | 110.33 | 2.14(1.60 to 2.69) | 4.93(3.46 to 6.90) | 129.68 |
| Fiji | 0.94(0.66 to 1.23) | 2.00(1.43 to 2.63) | 113.48 | 3.77(2.99 to 4.77) | 8.21(6.38 to 10.43) | 117.74 | 4.70(3.89 to 5.77) | 10.20(8.27 to 12.57) | 116.89 |
| Gabon | 0.48(0.35 to 0.65) | 1.34(0.94 to 1.76) | 176.54 | 5.42(4.01 to 6.81) | 9.66(6.21 to 12.90) | 78.32 | 5.90(4.46 to 7.30) | 11.00(7.46 to 14.30) | 86.39 |
| Georgia | 3.86(2.73 to 5.06) | 4.56(3.32 to 6.02) | 18.00 | 10.07(8.86 to 11.31) | 15.42(12.94 to 18.10) | 53.05 | 13.93(12.27 to 15.57) | 19.97(17.16 to 22.98) | 43.34 |
| Grenada | 0.10(0.08 to 0.14) | 0.25(0.18 to 0.34) | 143.48 | 0.61(0.55 to 0.67) | 1.24(1.09 to 1.40) | 104.74 | 0.71(0.64 to 0.78) | 1.50(1.32 to 1.67) | 110.40 |
| Guatemala | 6.60(4.66 to 8.82) | 30.73(21.60 to 41.29) | 365.34 | 40.23(35.98 to 44.56) | 158.42(124.40 to 200.57) | 293.78 | 46.83(42.43 to 52.01) | 189.15(153.23 to 232.94) | 303.87 |
| Guyana | 0.76(0.55 to 1.00) | 1.56(1.10 to 2.04) | 104.66 | 3.52(3.02 to 4.02) | 8.26(6.29 to 10.72) | 134.63 | 4.28(3.75 to 4.85) | 9.82(7.84 to 12.24) | 129.30 |
| Iran (Islamic Republic of) | 41.49(29.56 to 54.62) | 98.43(71.36 to 129.30) | 137.23 | 175.94(151.45 to 199.00) | 258.59(240.96 to 273.71) | 46.98 | 217.42(190.84 to 243.77) | 357.02(324.21 to 390.69) | 64.20 |
| Iraq | 14.34(10.44 to 18.80) | 56.85(40.98 to 74.91) | 296.51 | 97.78(78.60 to 124.88) | 194.42(147.23 to 252.09) | 98.84 | 112.12(93.08 to 140.11) | 251.27(199.05 to 310.08) | 124.11 |
| Jamaica | 2.56(1.82 to 3.38) | 5.62(4.03 to 7.53) | 119.71 | 9.24(8.64 to 9.83) | 21.89(17.10 to 27.60) | 136.85 | 11.80(10.89 to 12.82) | 27.52(22.44 to 33.63) | 133.14 |
| Jordan | 2.48(1.75 to 3.25) | 14.33(10.10 to 18.94) | 478.70 | 12.11(10.42 to 14.01) | 42.67(35.43 to 50.59) | 252.33 | 14.59(12.74 to 16.70) | 57.00(48.72 to 66.25) | 290.75 |
| Kazakhstan | 11.21(7.86 to 14.81) | 18.54(13.22 to 24.22) | 65.36 | 35.58(33.35 to 38.66) | 50.19(43.34 to 57.49) | 41.08 | 46.79(42.82 to 51.71) | 68.73(60.15 to 77.91) | 46.90 |
| Lebanon | 2.58(1.86 to 3.43) | 7.99(5.76 to 10.59) | 209.74 | 14.72(12.33 to 17.63) | 21.94(15.86 to 28.82) | 49.03 | 17.30(14.76 to 20.38) | 29.92(23.33 to 37.55) | 72.99 |
| Libya | 2.75(1.96 to 3.65) | 9.05(6.57 to 11.93) | 229.63 | 13.79(10.82 to 16.96) | 31.52(21.36 to 42.39) | 128.51 | 16.54(13.39 to 19.83) | 40.58(29.85 to 52.15) | 145.31 |
| Malaysia | 13.45(9.50 to 17.75) | 47.72(34.08 to 63.11) | 254.80 | 61.70(57.26 to 66.78) | 145.56(115.16 to 180.30) | 135.94 | 75.15(69.10 to 81.64) | 193.29(159.88 to 233.66) | 157.21 |
| Maldives | 0.15(0.11 to 0.20) | 0.65(0.44 to 0.87) | 323.24 | 1.42(1.20 to 1.73) | 2.04(1.71 to 2.38) | 42.86 | 1.58(1.36 to 1.89) | 2.69(2.30 to 3.11) | 70.19 |
| Marshall Islands | 0.04(0.03 to 0.05) | 0.10(0.07 to 0.13) | 148.37 | 0.21(0.17 to 0.27) | 0.56(0.40 to 0.77) | 160.60 | 0.25(0.21 to 0.32) | 0.66(0.50 to 0.87) | 158.70 |
| Mauritius | 1.52(1.08 to 1.99) | 4.52(3.21 to 6.04) | 198.48 | 8.58(8.11 to 9.09) | 30.17(24.40 to 37.04) | 251.69 | 10.09(9.47 to 10.79) | 34.69(28.60 to 41.77) | 243.70 |
| Mexico | 109.53(77.24 to 146.48) | 368.53(255.04 to 494.07) | 236.48 | 338.50(329.22 to 346.83) | 1774.47(1519.55 to 2032.33) | 424.21 | 448.03(414.93 to 485.34) | 2143.00(1871.57 to 2437.83) | 378.32 |
| Montenegro | 0.45(0.32 to 0.60) | 0.75(0.53 to 1.01) | 65.92 | 1.73(1.58 to 1.94) | 2.51(2.10 to 2.97) | 44.97 | 2.18(1.97 to 2.45) | 3.26(2.79 to 3.80) | 49.30 |
| Namibia | 0.67(0.48 to 0.90) | 1.65(1.18 to 2.22) | 146.92 | 4.84(3.41 to 6.71) | 8.73(5.93 to 12.42) | 80.30 | 5.51(4.08 to 7.38) | 10.38(7.48 to 14.02) | 88.39 |
| Nauru | 0.01(0.01 to 0.02) | 0.02(0.01 to 0.03) | 60.97 | 0.07(0.05 to 0.09) | 0.10(0.07 to 0.13) | 47.22 | 0.08(0.06 to 0.10) | 0.12(0.09 to 0.15) | 49.23 |
| North Macedonia | 1.40(0.99 to 1.85) | 2.82(1.99 to 3.83) | 100.99 | 5.01(4.60 to 5.53) | 7.66(6.05 to 9.76) | 53.04 | 6.41(5.84 to 7.11) | 10.48(8.64 to 12.75) | 63.53 |
| Paraguay | 2.54(1.82 to 3.37) | 8.30(5.94 to 11.07) | 226.77 | 8.89(8.00 to 9.87) | 43.92(33.38 to 56.55) | 393.89 | 11.43(10.21 to 12.63) | 52.21(41.44 to 65.89) | 356.77 |
| Peru | 11.04(7.75 to 14.79) | 36.67(26.27 to 49.42) | 232.22 | 88.48(78.65 to 99.51) | 160.93(118.87 to 211.56) | 81.88 | 99.52(89.02 to 110.86) | 197.60(154.35 to 251.28) | 98.56 |
| Romania | 15.56(11.05 to 20.82) | 27.43(19.65 to 36.37) | 76.30 | 67.00(64.38 to 69.50) | 56.19(46.37 to 67.48) | -16.13 | 82.56(77.29 to 88.00) | 83.62(70.86 to 98.18) | 1.28 |
| Russian Federation | 124.88(88.79 to 166.85) | 162.77(116.30 to 212.83) | 30.34 | 290.36(284.31 to 301.27) | 261.36(228.56 to 295.87) | -9.99 | 415.24(378.96 to 456.12) | 424.13(367.95 to 487.35) | 2.14 |
| Saint Lucia | 0.14(0.10 to 0.19) | 0.41(0.29 to 0.54) | 181.95 | 0.69(0.64 to 0.75) | 1.69(1.41 to 2.02) | 144.36 | 0.84(0.77 to 0.91) | 2.10(1.80 to 2.45) | 150.84 |
| Saint Vincent and the Grenadines | 0.12(0.08 to 0.15) | 0.26(0.18 to 0.34) | 121.80 | 0.47(0.43 to 0.52) | 1.13(0.96 to 1.31) | 137.81 | 0.59(0.53 to 0.64) | 1.38(1.19 to 1.60) | 134.68 |
| Samoa | 0.17(0.13 to 0.23) | 0.38(0.27 to 0.50) | 114.81 | 0.89(0.68 to 1.16) | 1.65(1.27 to 2.13) | 84.84 | 1.07(0.84 to 1.35) | 2.03(1.61 to 2.51) | 89.75 |
| Serbia | 6.83(4.85 to 8.99) | 10.89(7.75 to 14.69) | 59.35 | 32.12(29.09 to 37.13) | 42.79(34.55 to 51.74) | 33.21 | 38.95(35.00 to 44.37) | 53.68(44.47 to 63.60) | 37.80 |
| South Africa | 24.92(17.76 to 32.87) | 59.42(42.16 to 78.39) | 138.48 | 144.37(131.89 to 158.63) | 307.03(282.98 to 330.82) | 112.67 | 169.28(155.90 to 185.02) | 366.45(337.30 to 397.22) | 116.47 |
| Sri Lanka | 14.79(10.61 to 19.33) | 40.20(28.54 to 53.03) | 171.72 | 72.34(63.68 to 79.86) | 127.03(93.86 to 168.38) | 75.59 | 87.14(77.54 to 95.87) | 167.23(132.49 to 211.29) | 91.91 |
| Suriname | 0.43(0.30 to 0.56) | 1.23(0.88 to 1.67) | 189.69 | 2.15(1.86 to 2.36) | 6.60(5.40 to 7.86) | 206.73 | 2.58(2.24 to 2.83) | 7.83(6.66 to 9.18) | 203.92 |
| Thailand | 62.06(44.69 to 83.06) | 149.66(109.64 to 198.43) | 141.16 | 306.59(266.34 to 353.39) | 571.10(429.87 to 741.58) | 86.27 | 368.65(327.38 to 423.07) | 720.75(577.00 to 904.74) | 95.51 |
| Tonga | 0.11(0.07 to 0.14) | 0.19(0.13 to 0.25) | 76.90 | 0.41(0.32 to 0.55) | 0.78(0.60 to 1.07) | 92.65 | 0.51(0.42 to 0.66) | 0.97(0.77 to 1.26) | 89.39 |
| Turkey | 42.01(30.65 to 55.46) | 122.41(88.21 to 163.82) | 191.41 | 315.46(268.27 to 389.43) | 357.13(288.13 to 437.79) | 13.21 | 357.46(307.68 to 434.85) | 479.54(402.78 to 569.18) | 34.15 |
| Turkmenistan | 2.35(1.63 to 3.12) | 5.51(3.89 to 7.36) | 134.90 | 12.63(11.74 to 13.53) | 25.08(20.20 to 31.42) | 98.54 | 14.98(13.87 to 16.17) | 30.59(25.30 to 37.33) | 104.23 |
| Tuvalu | 0.01(0.01 to 0.01) | 0.02(0.02 to 0.03) | 104.72 | 0.08(0.06 to 0.10) | 0.13(0.09 to 0.17) | 62.17 | 0.09(0.07 to 0.11) | 0.15(0.11 to 0.20) | 67.48 |
| Venezuela (Bolivarian Republic of) | 18.25(12.92 to 24.41) | 57.99(40.25 to 78.32) | 217.76 | 44.27(42.10 to 46.56) | 243.05(181.14 to 318.99) | 4.49(3.05 to 6.29) | 62.52(56.51 to 69.13) | 301.05(235.11 to 381.74) | 381.50 |

*CKD* Chronic kidney disease, *YLD* Years lived with disability, *YLL* Years of life lost, *DALY* Disability-adjusted life-years, *LICs* Low-income countries, *Lower* *MICs* Lower-middle income countries, *Upper MICs* Upper-middle income countries.

Table S2. Age-standardized YLD, YLL and DALY rates of CKD with percent change in 137 low-and middle-income countries, 1990-2019

| country | Age-standardized YLD rate per 100 000 persons | | | Age-standardized YLL rate per 100 000 persons | | | Age-standardized DALY rate per 100 000 persons | | |
| --- | --- | --- | --- | --- | --- | --- | --- | --- | --- |
|  | 1990 | 2019 | % change | 1990 | 2019 | % change | 1990 | 2019 | % change |
| **LICs** |  |  |  |  |  |  |  |  |  |
| Afghanistan | 103.33(75.78 to 135.41) | 152.46(111.77 to 202.44) | 47.54 | 1333.56(1024.72 to 1745.66) | 1077.91(821.64 to 1523.53) | -19.17 | 1436.89(1121.17 to 1871.28) | 1230.37(958.38 to 1680.46) | -14.37 |
| Benin | 63.26(46.34 to 84.64) | 85.86(62.51 to 112.78) | 35.72 | 832.72(707.92 to 970.13) | 738.92(554.21 to 973.99) | -11.26 | 895.98(766.75 to 1033.96) | 824.78(630.43 to 1067.96) | -7.95 |
| Burkina Faso | 57.18(41.45 to 76.08) | 82.46(60.03 to 110.35) | 44.21 | 691.55(568.23 to 824.14) | 717.10(575.29 to 877.63) | 3.69 | 748.73(619.90 to 881.34) | 799.55(651.75 to 952.28) | 6.79 |
| Burundi | 58.53(42.08 to 78.22) | 68.32(48.91 to 90.47) | 16.73 | 749.16(583.95 to 958.28) | 551.88(426.97 to 704.43) | -26.33 | 807.69(643.78 to 1017.12) | 620.20(489.59 to 775.78) | -23.21 |
| Central African Republic | 48.49(34.36 to 64.65) | 62.68(44.42 to 82.70) | 29.26 | 803.01(609.19 to 998.47) | 777.99(576.84 to 1028.17) | -3.12 | 851.50(657.31 to 1046.93) | 840.67(634.11 to 1094.27) | -1.27 |
| Chad | 57.40(41.92 to 75.52) | 75.29(54.23 to 100.04) | 31.16 | 758.93(580.27 to 1058.07) | 734.85(543.23 to 973.90) | -3.17 | 816.33(636.61 to 1112.09) | 810.14(618.31 to 1045.62) | -0.76 |
| Democratic People's Republic of Korea | 116.02(84.27 to 152.81) | 132.31(96.19 to 172.84) | 14.04 | 421.66(322.64 to 534.51) | 355.50(279.45 to 447.05) | -15.69 | 537.69(434.95 to 654.44) | 487.81(399.40 to 587.41) | -9.28 |
| Democratic Republic of the Congo | 52.17(37.49 to 69.59) | 64.11(46.45 to 85.19) | 22.89 | 708.49(590.97 to 836.60) | 548.50(434.12 to 681.45) | -22.58 | 760.66(638.24 to 890.83) | 612.61(496.50 to 743.01) | -19.46 |
| Eritrea | 56.81(40.47 to 75.97) | 77.90(54.97 to 104.08) | 37.13 | 618.85(416.97 to 859.38) | 623.66(426.89 to 873.73) | 0.78 | 675.66(467.60 to 914.20) | 701.57(504.19 to 941.09) | 3.83 |
| Ethiopia | 58.54(41.75 to 76.83) | 70.30(50.58 to 93.17) | 20.09 | 982.00(803.15 to 1162.01) | 522.69(454.91 to 600.02) | -46.77 | 1040.53(860.30 to 1224.07) | 592.99(523.05 to 674.12) | -43.01 |
| Gambia | 61.82(44.70 to 81.78) | 83.84(60.71 to 110.34) | 35.60 | 708.98(532.03 to 932.23) | 723.69(563.43 to 923.67) | 2.07 | 770.81(592.21 to 993.93) | 807.52(635.46 to 1007.36) | 4.76 |
| Guinea | 64.04(46.22 to 84.68) | 84.37(61.54 to 112.69) | 31.74 | 921.54(745.85 to 1184.77) | 815.08(630.40 to 1031.17) | -11.55 | 985.58(805.75 to 1243.09) | 899.45(710.76 to 1120.95) | -8.74 |
| Guinea-Bissau | 71.02(50.86 to 94.20) | 91.57(66.09 to 119.11) | 28.95 | 1310.02(1021.27 to 1633.86) | 984.50(768.14 to 1225.99) | -24.85 | 1381.04(1095.13 to 1703.29) | 1076.07(855.94 to 1324.91) | -22.08 |
| Haiti | 121.51(87.40 to 160.02) | 166.10(120.32 to 218.94) | 36.69 | 954.35(746.78 to 1330.70) | 877.14(618.42 to 1327.13) | -8.09 | 1075.87(864.06 to 1432.46) | 1043.24(770.41 to 1508.47) | -3.03 |
| Liberia | 64.36(46.33 to 85.34) | 81.76(59.66 to 108.13) | 27.03 | 1016.07(835.45 to 1242.11) | 733.38(529.41 to 1014.86) | -27.82 | 1080.43(894.95 to 1310.50) | 815.14(610.14 to 1095.49) | -24.55 |
| Madagascar | 57.23(41.54 to 76.92) | 69.98(50.38 to 92.94) | 22.29 | 555.45(435.79 to 692.71) | 444.72(341.77 to 581.42) | -19.94 | 612.68(493.19 to 744.56) | 514.70(408.65 to 659.13) | -15.99 |
| Malawi | 62.44(44.83 to 82.85) | 80.40(57.46 to 107.19) | 28.76 | 642.97(523.06 to 746.86) | 562.36(457.76 to 681.38) | -12.54 | 705.41(588.88 to 806.29) | 642.77(534.27 to 761.07) | -8.88 |
| Mali | 63.73(46.32 to 83.97) | 82.64(60.14 to 109.28) | 29.67 | 928.67(735.70 to 1172.53) | 728.01(555.47 to 949.06) | -21.61 | 992.40(798.02 to 1236.02) | 810.65(635.31 to 1032.21) | -18.31 |
| Mozambique | 55.26(40.00 to 73.68) | 80.28(57.76 to 106.63) | 45.28 | 516.85(420.28 to 635.80) | 568.99(445.27 to 714.92) | 10.09 | 572.11(476.10 to 692.27) | 649.27(520.54 to 795.58) | 13.49 |
| Nepal | 75.19(53.80 to 98.83) | 121.72(88.02 to 159.38) | 61.87 | 481.99(354.81 to 626.96) | 575.77(400.49 to 762.17) | 19.46 | 557.18(432.10 to 700.34) | 697.49(522.11 to 897.63) | 25.18 |
| Niger | 70.49(50.66 to 92.83) | 85.19(62.11 to 111.85) | 20.84 | 880.02(697.09 to 1109.46) | 655.82(482.56 to 865.91) | -25.48 | 950.52(765.96 to 1179.39) | 741.00(567.80 to 950.58) | -22.04 |
| Rwanda | 61.15(43.80 to 81.09) | 75.33(53.82 to 100.60) | 23.18 | 784.97(642.46 to 931.89) | 510.48(398.20 to 632.64) | -34.97 | 846.12(700.66 to 994.74) | 585.80(469.84 to 712.56) | -30.77 |
| Sierra Leone | 61.03(44.39 to 80.98) | 82.40(59.29 to 108.24) | 35.02 | 784.20(610.67 to 981.84) | 674.06(510.91 to 881.81) | -14.04 | 845.23(672.65 to 1044.91) | 756.46(584.94 to 965.44) | -10.50 |
| Somalia | 63.37(45.74 to 84.42) | 76.66(54.88 to 101.51) | 20.96 | 744.88(564.12 to 937.11) | 685.44(519.32 to 912.42) | -7.98 | 808.25(629.96 to 1006.92) | 762.10(593.39 to 984.14) | -5.71 |
| South Sudan | 56.55(40.95 to 75.20) | 70.94(50.89 to 93.03) | 25.45 | 619.70(453.46 to 803.66) | 570.64(415.00 to 770.70) | -7.92 | 676.25(509.33 to 861.21) | 641.58(477.47 to 838.84) | -5.13 |
| Syrian Arab Republic | 117.65(85.55 to 155.17) | 149.50(108.97 to 196.71) | 27.07 | 837.42(689.93 to 1021.58) | 535.35(417.39 to 690.96) | -36.07 | 955.07(803.08 to 1143.63) | 684.86(556.83 to 836.76) | -28.29 |
| Tajikistan | 57.53(41.07 to 76.13) | 81.55(57.63 to 106.02) | 41.74 | 95.33(88.17 to 104.67) | 216.62(177.21 to 264.49) | 127.23 | 152.86(135.43 to 172.03) | 298.16(252.87 to 350.40) | 95.06 |
| Togo | 67.35(48.65 to 90.00) | 86.91(62.47 to 114.88) | 29.04 | 800.66(644.29 to 1010.71) | 689.13(536.62 to 873.81) | -13.93 | 868.01(711.27 to 1073.52) | 776.04(622.58 to 967.26) | -10.60 |
| Uganda | 52.78(37.82 to 70.37) | 68.04(49.00 to 90.04) | 28.92 | 540.07(413.05 to 686.09) | 539.74(416.85 to 681.73) | -0.06 | 592.85(466.71 to 734.40) | 607.78(483.98 to 746.93) | 2.52 |
| United Republic of Tanzania | 64.36(46.14 to 84.92) | 90.42(66.12 to 121.62) | 40.50 | 595.93(498.64 to 716.73) | 577.53(479.44 to 685.40) | -3.09 | 660.29(560.78 to 777.09) | 667.95(561.52 to 790.01) | 1.16 |
| Yemen | 87.84(64.03 to 115.54) | 134.99(97.73 to 177.95) | 53.67 | 553.40(411.93 to 747.83) | 476.57(362.33 to 632.43) | -13.88 | 641.24(494.49 to 835.55) | 611.56(486.80 to 769.32) | -4.63 |
| **Lower MICs** |  |  |  |  |  |  |  |  |  |
| Angola | 45.01(32.36 to 60.01) | 64.10(45.62 to 84.85) | 42.43 | 671.70(483.58 to 845.47) | 545.15(381.66 to 697.80) | -18.84 | 716.70(535.08 to 888.93) | 609.26(440.86 to 763.81) | -14.99 |
| Bangladesh | 53.88(38.97 to 71.51) | 81.29(58.82 to 106.85) | 50.87 | 390.55(259.01 to 494.60) | 263.21(214.10 to 318.81) | -32.60 | 444.43(309.94 to 550.10) | 344.51(288.14 to 407.74) | -22.48 |
| Bhutan | 79.65(57.33 to 106.08) | 117.11(84.72 to 153.65) | 47.03 | 572.97(372.90 to 792.02) | 624.26(437.39 to 808.81) | 8.95 | 652.62(453.70 to 870.23) | 741.37(555.09 to 934.40) | 13.60 |
| Bolivia (Plurinational State of) | 82.25(59.57 to 109.76) | 112.86(80.82 to 150.09) | 37.22 | 932.01(770.88 to 1149.17) | 1114.29(847.73 to 1420.82) | 19.56 | 1014.26(855.54 to 1226.94) | 1227.15(956.53 to 1537.15) | 20.99 |
| Cabo Verde | 53.80(38.38 to 71.20) | 80.70(58.23 to 108.26) | 50.01 | 377.29(341.57 to 426.82) | 491.33(423.46 to 566.16) | 30.23 | 431.08(390.71 to 480.86) | 572.04(497.93 to 647.97) | 32.70 |
| Cambodia | 89.30(64.31 to 116.19) | 123.44(88.92 to 162.99) | 38.24 | 670.79(552.13 to 800.42) | 484.96(390.56 to 581.04) | -27.70 | 760.09(640.98 to 887.19) | 608.40(510.70 to 714.18) | -19.96 |
| Cameroon | 82.87(59.75 to 108.90) | 120.70(86.91 to 160.48) | 45.65 | 1062.20(817.83 to 1311.27) | 955.77(702.00 to 1263.71) | -10.02 | 1145.07(898.76 to 1394.02) | 1076.47(817.47 to 1393.59) | -5.99 |
| Comoros | 67.82(48.93 to 90.00) | 84.83(60.93 to 112.21) | 25.08 | 626.94(336.84 to 831.74) | 545.73(423.47 to 676.35) | -12.95 | 694.77(404.24 to 901.28) | 630.56(506.43 to 764.34) | -9.24 |
| Congo | 58.51(42.12 to 77.86) | 76.77(55.13 to 101.06) | 31.20 | 881.33(621.57 to 1121.59) | 673.95(465.44 to 886.30) | -23.53 | 939.84(680.25 to 1178.96) | 750.72(535.86 to 971.33) | -20.12 |
| Côte d'Ivoire | 75.46(54.52 to 99.79) | 98.00(71.19 to 127.34) | 29.88 | 932.71(760.36 to 1109.42) | 779.93(586.71 to 984.31) | -16.38 | 1008.17(836.58 to 1179.88) | 877.93(683.81 to 1090.72) | -12.92 |
| Djibouti | 60.86(43.93 to 80.36) | 84.92(61.23 to 113.07) | 39.53 | 520.22(378.99 to 689.46) | 590.95(446.30 to 800.18) | 13.60 | 581.08(439.90 to 752.91) | 675.87(527.45 to 888.01) | 16.31 |
| Egypt | 108.45(79.97 to 144.20) | 172.44(126.24 to 228.81) | 59.00 | 807.90(569.11 to 974.64) | 837.02(493.06 to 1200.31) | 3.60 | 916.35(675.75 to 1090.29) | 1009.46(660.38 to 1386.31) | 10.16 |
| El Salvador | 138.13(96.97 to 184.91) | 242.40(167.10 to 324.12) | 75.49 | 537.32(505.14 to 573.52) | 1668.55(1249.34 to 2200.92) | 210.53 | 675.44(622.51 to 737.20) | 1910.95(1484.58 to 2461.33) | 182.92 |
| Eswatini | 93.80(65.75 to 125.30) | 130.33(94.03 to 172.36) | 38.94 | 846.09(664.25 to 1035.69) | 1184.62(831.76 to 1578.32) | 40.01 | 939.90(756.12 to 1137.52) | 1314.95(959.32 to 1698.25) | 39.90 |
| Ghana | 59.86(43.59 to 79.88) | 88.08(64.34 to 115.41) | 47.13 | 657.70(489.74 to 881.69) | 710.16(510.51 to 911.42) | 7.98 | 717.57(543.50 to 938.25) | 798.23(597.45 to 1002.46) | 11.24 |
| Honduras | 132.03(93.27 to 176.96) | 183.51(131.71 to 244.78) | 38.99 | 660.02(530.85 to 883.31) | 1223.98(977.72 to 1590.88) | 85.45 | 792.05(656.64 to 1014.02) | 1407.49(1152.53 to 1787.96) | 77.70 |
| India | 104.83(75.94 to 136.63) | 107.07(77.58 to 139.60) | 2.14 | 550.93(471.62 to 621.55) | 505.49(435.76 to 583.89) | -8.25 | 655.76(570.21 to 734.75) | 612.57(535.56 to 691.20) | -6.59 |
| Indonesia | 96.63(68.79 to 127.99) | 136.71(98.00 to 180.93) | 41.48 | 617.04(552.91 to 685.95) | 536.70(460.60 to 622.44) | -13.02 | 713.68(648.14 to 788.40) | 673.41(588.41 to 764.67) | -5.64 |
| Kenya | 54.03(38.90 to 72.31) | 71.34(51.05 to 95.02) | 32.04 | 435.60(363.50 to 525.68) | 516.10(431.11 to 613.08) | 18.48 | 489.63(416.02 to 584.82) | 587.44(500.48 to 687.26) | 19.98 |
| Kiribati | 164.32(117.83 to 217.00) | 225.22(162.06 to 294.55) | 37.06 | 1218.41(993.85 to 1478.43) | 1481.27(1109.56 to 1924.43) | 21.57 | 1382.73(1152.87 to 1652.86) | 1706.49(1308.14 to 2159.50) | 23.41 |
| Kyrgyzstan | 84.14(59.39 to 112.37) | 96.49(68.47 to 128.63) | 14.68 | 463.22(420.57 to 508.25) | 302.83(262.74 to 347.50) | -34.62 | 547.35(495.75 to 596.78) | 399.32(349.69 to 454.67) | -27.05 |
| Lao People's Democratic Republic | 116.92(84.06 to 152.34) | 163.09(117.51 to 215.45) | 39.49 | 1379.83(1083.83 to 1720.78) | 991.45(751.94 to 1266.89) | -28.15 | 1496.75(1203.72 to 1838.68) | 1154.54(913.57 to 1438.63) | -22.86 |
| Lesotho | 72.51(51.74 to 97.06) | 106.87(76.47 to 144.25) | 47.39 | 560.40(448.90 to 698.73) | 1139.42(802.17 to 1511.31) | 103.32 | 632.91(517.71 to 770.04) | 1246.29(893.24 to 1617.35) | 96.91 |
| Mauritania | 72.58(51.70 to 96.54) | 93.64(67.34 to 123.35) | 29.01 | 1083.78(895.76 to 1278.45) | 633.93(458.21 to 837.72) | -41.51 | 1156.36(967.62 to 1351.57) | 727.57(545.42 to 937.31) | -37.08 |
| Micronesia (Federated States of) | 173.24(124.32 to 225.97) | 267.73(193.14 to 350.39) | 54.55 | 1269.84(994.30 to 1638.74) | 1895.00(1313.85 to 2478.01) | 49.23 | 1443.08(1169.69 to 1787.69) | 2162.73(1584.61 to 2761.55) | 49.87 |
| Mongolia | 92.04(65.74 to 121.80) | 108.04(77.88 to 142.69) | 17.38 | 967.90(821.57 to 1136.76) | 455.48(359.84 to 580.73) | -52.94 | 1059.94(908.24 to 1231.62) | 563.52(460.67 to 689.92) | -46.83 |
| Morocco | 88.44(63.93 to 117.39) | 165.15(119.26 to 219.06) | 86.73 | 617.54(531.48 to 776.40) | 679.42(523.68 to 859.06) | 10.02 | 705.98(615.60 to 865.71) | 844.57(673.16 to 1035.89) | 19.63 |
| Myanmar | 121.04(86.86 to 160.92) | 171.16(124.07 to 224.31) | 41.42 | 927.79(714.96 to 1161.62) | 692.59(559.27 to 859.96) | -25.35 | 1048.82(829.69 to 1278.99) | 863.75(719.80 to 1035.50) | -17.65 |
| Nicaragua | 157.78(111.63 to 212.15) | 248.69(171.44 to 337.46) | 57.61 | 945.93(884.67 to 1016.45) | 1863.29(1517.55 to 2232.95) | 96.98 | 1103.72(1025.57 to 1187.91) | 2111.98(1753.72 to 2500.72) | 91.35 |
| Nigeria | 61.95(45.06 to 82.07) | 84.42(61.90 to 111.26) | 36.27 | 639.04(510.85 to 823.13) | 528.25(413.49 to 665.93) | -17.34 | 700.99(573.23 to 888.72) | 612.67(495.43 to 755.18) | -12.60 |
| Pakistan | 89.47(64.47 to 118.28) | 123.22(88.71 to 161.20) | 37.73 | 667.82(498.16 to 895.72) | 1033.13(757.41 to 1319.47) | 54.70 | 757.29(588.29 to 986.18) | 1156.35(873.19 to 1447.54) | 52.70 |
| Palestine | 122.99(90.23 to 161.34) | 168.49(124.52 to 224.13) | 37.00 | 970.46(768.31 to 1204.23) | 641.43(547.17 to 737.03) | -33.90 | 1093.45(884.26 to 1330.85) | 809.92(707.33 to 918.09) | -25.93 |
| Papua New Guinea | 90.37(64.65 to 119.12) | 122.42(87.41 to 161.34) | 35.47 | 358.70(287.04 to 437.62) | 406.50(309.87 to 522.88) | 13.32 | 449.07(374.60 to 528.48) | 528.91(425.10 to 653.43) | 17.78 |
| Philippines | 109.47(79.07 to 143.61) | 169.93(122.21 to 225.39) | 55.23 | 873.35(792.36 to 962.58) | 1109.56(925.56 to 1317.02) | 27.05 | 982.82(897.69 to 1079.19) | 1279.50(1090.34 to 1488.44) | 30.19 |
| Republic of Moldova | 44.12(31.75 to 59.04) | 61.53(43.43 to 81.20) | 39.46 | 60.47(57.13 to 63.74) | 112.91(97.93 to 128.41) | 86.71 | 104.60(91.49 to 119.37) | 174.44(152.02 to 199.25) | 66.78 |
| Saotome and Principe | 82.41(59.71 to 108.86) | 110.64(79.51 to 146.61) | 34.26 | 1018.17(797.24 to 1188.62) | 1107.92(877.52 to 1332.74) | 8.81 | 1100.58(878.03 to 1269.76) | 1218.56(983.37 to 1449.41) | 10.72 |
| Senegal | 65.24(47.15 to 86.55) | 77.55(55.95 to 102.46) | 18.88 | 968.58(745.26 to 1245.46) | 764.59(572.23 to 1025.89) | -21.06 | 1033.81(805.56 to 1320.03) | 842.15(643.58 to 1100.46) | -18.54 |
| Solomon Islands | 174.46(125.12 to 229.09) | 210.21(150.00 to 275.54) | 20.49 | 1316.80(988.68 to 1768.23) | 765.66(609.17 to 937.79) | -41.85 | 1491.26(1150.45 to 1941.82) | 975.87(812.34 to 1155.90) | -34.56 |
| Sudan | 84.17(61.26 to 111.66) | 137.64(100.02 to 183.35) | 63.53 | 653.80(517.32 to 856.39) | 579.67(409.08 to 865.65) | -11.34 | 737.96(596.02 to 942.15) | 717.30(535.03 to 1013.09) | -2.80 |
| Timor-Leste | 92.20(66.97 to 119.42) | 130.40(95.06 to 170.40) | 41.44 | 791.87(605.29 to 1050.43) | 702.59(501.23 to 911.30) | -11.27 | 884.07(702.71 to 1142.55) | 833.00(612.76 to 1049.22) | -5.78 |
| Tunisia | 94.17(68.24 to 124.38) | 142.45(103.66 to 189.24) | 51.27 | 483.01(414.93 to 566.70) | 426.12(304.37 to 571.15) | -11.78 | 577.18(504.01 to 666.43) | 568.57(441.00 to 718.86) | -1.49 |
| Ukraine | 43.30(30.60 to 58.02) | 56.53(39.81 to 75.38) | 30.57 | 42.50(40.65 to 44.25) | 100.16(84.22 to 118.21) | 135.68 | 85.80(72.28 to 100.37) | 156.69(132.82 to 183.41) | 82.63 |
| Uzbekistan | 97.99(70.34 to 128.82) | 130.42(93.63 to 172.42) | 33.10 | 459.38(403.18 to 597.45) | 539.97(457.92 to 630.59) | 17.54 | 557.37(490.44 to 694.57) | 670.40(576.62 to 771.85) | 20.28 |
| Vanuatu | 126.88(91.48 to 167.50) | 183.97(131.58 to 239.87) | 44.99 | 634.17(440.25 to 870.38) | 1068.55(777.77 to 1431.22) | 68.50 | 761.05(562.05 to 1007.74) | 1252.52(947.51 to 1628.46) | 64.58 |
| Viet Nam | 85.03(61.74 to 112.31) | 117.00(84.24 to 155.34) | 37.60 | 638.76(515.52 to 794.25) | 534.89(416.55 to 652.95) | -16.26 | 723.80(598.33 to 879.47) | 651.90(527.73 to 779.63) | -9.93 |
| Zambia | 71.84(51.87 to 95.32) | 99.27(71.67 to 132.17) | 38.18 | 768.69(652.26 to 898.02) | 674.94(539.17 to 842.01) | -12.20 | 840.53(718.21 to 970.85) | 774.21(632.41 to 938.65) | -7.89 |
| Zimbabwe | 75.11(54.30 to 99.14) | 99.43(70.61 to 130.38) | 32.37 | 547.71(415.24 to 809.86) | 816.27(585.13 to 1195.05) | 49.03 | 622.82(487.26 to 894.65) | 915.70(683.94 to 1309.60) | 47.02 |
| **Upper MICs** |  |  |  |  |  |  |  |  |  |
| Albania | 60.71(43.67 to 78.97) | 78.02(55.73 to 104.17) | 28.52 | 297.85(278.15 to 320.51) | 200.23(152.77 to 259.86) | -32.78 | 358.56(331.93 to 388.01) | 278.25(221.25 to 342.24) | -22.40 |
| Algeria | 103.13(75.42 to 137.36) | 157.28(113.64 to 209.64) | 52.50 | 736.53(573.89 to 1018.10) | 544.25(424.32 to 720.35) | -26.11 | 839.66(668.56 to 1130.07) | 701.53(567.99 to 889.89) | -16.45 |
| American Samoa | 167.34(121.80 to 221.87) | 250.49(179.78 to 327.71) | 49.69 | 904.49(764.84 to 1039.34) | 1346.14(1129.46 to 1610.64) | 48.83 | 1071.83(920.69 to 1215.77) | 1596.63(1364.49 to 1879.91) | 48.96 |
| Argentina | 88.02(63.97 to 115.21) | 94.24(68.92 to 122.79) | 7.07 | 480.49(458.46 to 503.61) | 511.10(475.63 to 551.45) | 6.37 | 568.51(535.61 to 602.96) | 605.35(559.42 to 653.29) | 6.48 |
| Armenia | 57.46(40.06 to 76.54) | 87.56(62.09 to 117.68) | 52.38 | 56.95(52.50 to 61.40) | 228.83(193.44 to 268.03) | 301.77 | 114.42(97.27 to 134.15) | 316.39(269.98 to 362.87) | 176.52 |
| Azerbaijan | 73.25(52.29 to 96.05) | 111.58(79.50 to 146.11) | 52.34 | 307.72(274.33 to 344.07) | 425.11(355.21 to 495.67) | 38.15 | 380.96(339.89 to 421.40) | 536.69(462.31 to 619.25) | 40.88 |
| Belarus | 46.40(33.08 to 62.92) | 59.12(41.38 to 79.36) | 27.41 | 42.05(39.78 to 44.19) | 69.79(55.13 to 89.34) | 65.97 | 88.46(74.53 to 105.00) | 128.92(104.56 to 156.45) | 45.74 |
| Belize | 134.06(96.47 to 175.32) | 213.04(152.23 to 279.94) | 58.91 | 545.03(494.36 to 597.15) | 1001.39(861.87 to 1161.41) | 83.73 | 679.09(614.67 to 749.31) | 1214.44(1059.15 to 1393.09) | 78.83 |
| Bosnia and Herzegovina | 63.75(46.14 to 83.37) | 87.81(63.35 to 117.19) | 37.74 | 211.14(199.05 to 223.47) | 219.76(173.97 to 274.20) | 4.08 | 274.90(251.42 to 298.59) | 307.57(255.56 to 367.75) | 11.89 |
| Botswana | 76.33(54.68 to 102.74) | 115.65(83.98 to 154.18) | 51.52 | 615.87(443.73 to 858.65) | 816.86(561.70 to 1118.16) | 32.63 | 692.20(512.58 to 929.53) | 932.50(673.13 to 1233.63) | 34.72 |
| Brazil | 97.64(70.05 to 127.89) | 113.88(82.55 to 150.08) | 16.63 | 464.93(447.16 to 479.72) | 390.35(369.67 to 408.70) | -16.04 | 562.57(530.66 to 595.93) | 504.23(465.82 to 542.52) | -10.37 |
| Bulgaria | 63.85(45.77 to 85.03) | 95.04(67.53 to 126.20) | 48.85 | 177.57(168.40 to 187.27) | 345.65(273.64 to 436.07) | 94.65 | 241.42(220.81 to 263.87) | 440.69(360.89 to 536.55) | 82.54 |
| China | 82.52(59.75 to 109.40) | 87.48(62.29 to 117.23) | 6.01 | 327.53(289.19 to 366.88) | 224.17(190.99 to 256.39) | -31.56 | 410.05(363.61 to 456.52) | 311.64(267.99 to 353.67) | -24.00 |
| Colombia | 118.02(84.15 to 158.08) | 146.83(103.00 to 199.72) | 24.41 | 477.85(454.45 to 500.04) | 354.59(274.80 to 454.69) | -25.80 | 595.87(553.70 to 638.69) | 501.42(407.07 to 617.49) | -15.85 |
| Costa Rica | 192.54(135.03 to 257.67) | 211.83(146.76 to 281.82) | 10.02 | 317.66(296.70 to 340.96) | 554.30(433.64 to 696.97) | 74.49 | 510.20(451.09 to 573.55) | 766.13(617.11 to 935.90) | 50.16 |
| Cuba | 92.23(65.71 to 123.10) | 134.95(96.47 to 179.62) | 46.32 | 211.04(201.87 to 221.16) | 336.97(272.02 to 412.06) | 59.67 | 303.27(275.20 to 334.75) | 471.92(394.86 to 561.77) | 55.61 |
| Dominica | 162.12(115.49 to 215.28) | 222.81(157.05 to 297.47) | 37.44 | 837.96(735.67 to 943.97) | 1113.90(897.15 to 1368.36) | 32.93 | 1000.08(886.76 to 1116.22) | 1336.71(1108.43 to 1619.83) | 33.66 |
| Dominican Republic | 92.28(66.58 to 122.52) | 146.86(105.63 to 194.61) | 59.15 | 392.47(349.90 to 438.73) | 612.68(444.56 to 831.38) | 56.11 | 484.74(431.99 to 541.05) | 759.53(585.72 to 979.84) | 56.69 |
| Ecuador | 80.88(57.17 to 108.61) | 141.69(100.99 to 191.24) | 75.18 | 479.54(452.99 to 508.58) | 967.32(769.29 to 1239.66) | 101.72 | 560.42(524.28 to 602.41) | 1109.00(899.22 to 1381.37) | 97.89 |
| Equatorial Guinea | 47.32(33.76 to 62.43) | 76.87(55.77 to 101.69) | 62.44 | 735.15(543.59 to 939.29) | 644.11(452.62 to 908.01) | -12.38 | 782.47(586.02 to 983.78) | 720.98(526.26 to 981.01) | -7.86 |
| Fiji | 173.55(124.64 to 226.42) | 237.74(171.80 to 311.17) | 36.99 | 819.44(658.55 to 1025.90) | 1050.14(826.64 to 1314.67) | 28.15 | 992.99(829.15 to 1221.33) | 1287.88(1048.66 to 1570.46) | 29.70 |
| Gabon | 65.33(47.38 to 87.29) | 95.62(68.76 to 124.82) | 46.36 | 818.98(599.92 to 1026.92) | 834.13(535.70 to 1093.89) | 1.85 | 884.31(658.64 to 1096.17) | 929.74(627.29 to 1198.08) | 5.14 |
| Georgia | 66.32(46.78 to 86.61) | 96.30(68.70 to 126.68) | 45.20 | 170.43(150.41 to 190.11) | 297.03(250.03 to 348.53) | 74.29 | 236.75(209.30 to 263.55) | 393.33(336.58 to 451.55) | 66.14 |
| Grenada | 143.08(102.12 to 191.13) | 227.05(163.57 to 301.49) | 58.68 | 862.11(777.63 to 947.11) | 1110.07(976.02 to 1240.79) | 28.76 | 1005.19(907.44 to 1101.74) | 1337.12(1186.74 to 1485.69) | 33.02 |
| Guatemala | 131.16(93.20 to 175.83) | 221.78(155.62 to 298.40) | 69.09 | 775.26(693.30 to 863.24) | 1276.81(1009.65 to 1606.32) | 64.69 | 906.42(819.05 to 1011.03) | 1498.59(1213.21 to 1841.56) | 65.33 |
| Guyana | 138.89(100.38 to 183.51) | 218.89(155.64 to 284.70) | 57.60 | 733.41(631.98 to 836.68) | 1196.23(918.29 to 1536.90) | 63.10 | 872.30(767.53 to 987.19) | 1415.11(1133.41 to 1756.76) | 62.23 |
| Iran(Islamic Republic of) | 112.90(82.68 to 148.10) | 126.21(91.53 to 165.66) | 11.78 | 507.44(463.24 to 556.81) | 360.47(333.67 to 382.16) | -28.96 | 620.34(569.14 to 676.86) | 486.68(443.93 to 530.46) | -21.55 |
| Iraq | 129.85(94.65 to 171.37) | 189.19(138.29 to 248.21) | 45.69 | 996.98(808.42 to 1279.53) | 802.01(615.85 to 1021.42) | -19.56 | 1126.83(933.87 to 1407.75) | 991.20(797.31 to 1215.04) | -12.04 |
| Jamaica | 132.48(94.77 to 175.51) | 187.45(134.29 to 251.12) | 41.49 | 496.85(464.25 to 529.30) | 731.08(570.64 to 922.27) | 47.14 | 629.33(581.35 to 683.86) | 918.54(748.81 to 1125.74) | 45.95 |
| Jordan | 118.45(86.10 to 156.32) | 172.74(125.07 to 230.74) | 45.83 | 780.95(661.23 to 912.00) | 653.07(545.61 to 774.73) | -16.38 | 899.41(777.58 to 1042.36) | 825.82(707.85 to 957.14) | -8.18 |
| Kazakhstan | 74.86(53.39 to 98.46) | 100.74(71.97 to 130.63) | 34.57 | 238.31(224.05 to 258.09) | 281.11(244.32 to 320.44) | 17.96 | 313.17(287.52 to 345.37) | 381.86(336.00 to 431.67) | 21.93 |
| Lebanon | 102.75(74.87 to 137.29) | 151.51(108.62 to 200.59) | 47.46 | 633.61(532.66 to 762.52) | 423.15(307.18 to 555.68) | -33.22 | 736.36(629.66 to 867.20) | 574.66(449.11 to 717.08) | -21.96 |
| Libya | 104.77(76.16 to 139.11) | 155.72(113.50 to 207.01) | 48.62 | 613.06(469.18 to 770.83) | 599.98(403.11 to 810.46) | -2.13 | 717.84(571.93 to 880.67) | 755.70(552.53 to 970.69) | 5.27 |
| Malaysia | 106.82(77.46 to 140.05) | 160.89(115.86 to 213.29) | 50.61 | 574.69(530.71 to 623.60) | 529.83(420.36 to 650.70) | -7.81 | 681.52(626.66 to 738.33) | 690.72(575.96 to 828.12) | 1.35 |
| Maldives | 116.20(84.42 to 151.22) | 153.90(109.72 to 205.32) | 32.45 | 1270.10(1104.01 to 1509.48) | 619.93(517.65 to 731.08) | -51.19 | 1386.30(1221.08 to 1632.20) | 773.83(659.66 to 893.21) | -44.18 |
| Marshall Islands | 147.14(106.48 to 194.20) | 209.15(150.89 to 273.55) | 42.14 | 986.31(786.45 to 1279.73) | 1324.61(955.29 to 1799.63) | 34.30 | 1133.45(927.88 to 1434.50) | 1533.75(1163.87 to 2020.13) | 35.32 |
| Mauritius | 167.59(122.11 to 219.54) | 282.14(199.81 to 372.29) | 68.35 | 1058.27(999.52 to 1122.31) | 1750.34(1424.29 to 2144.26) | 65.40 | 1225.86(1148.02 to 1307.73) | 2032.48(1683.03 to 2441.10) | 65.80 |
| Mexico | 190.06(135.53 to 254.18) | 296.40(206.99 to 394.46) | 55.95 | 651.71(629.81 to 668.16) | 1469.06(1261.50 to 1680.10) | 125.42 | 841.77(783.21 to 909.62) | 1765.46(1544.91 to 2006.16) | 109.73 |
| Montenegro | 71.11(50.22 to 94.83) | 89.26(63.23 to 118.87) | 25.52 | 279.50(254.70 to 312.43) | 277.12(232.08 to 326.92) | -0.85 | 350.62(317.29 to 392.73) | 366.38(316.23 to 425.05) | 4.50 |
| Namibia | 70.86(50.63 to 94.78) | 91.88(66.06 to 123.81) | 29.68 | 607.66(436.55 to 836.99) | 567.80(400.58 to 802.74) | -6.56 | 678.52(506.55 to 911.52) | 659.69(486.03 to 888.71) | -2.78 |
| Nauru | 177.26(126.24 to 233.43) | 257.27(186.63 to 342.38) | 45.14 | 1238.61(926.24 to 1549.50) | 1647.93(1150.34 to 2103.76) | 33.05 | 1415.87(1101.28 to 1735.07) | 1905.20(1405.14 to 2364.18) | 34.56 |
| North Macedonia | 70.99(50.08 to 93.66) | 99.32(70.83 to 132.53) | 39.91 | 262.21(241.81 to 290.43) | 252.89(201.44 to 318.35) | -3.56 | 333.21(303.16 to 369.41) | 352.21(292.29 to 425.00) | 5.70 |
| Paraguay | 89.17(64.21 to 117.29) | 133.26(95.61 to 178.19) | 49.46 | 344.53(308.24 to 381.62) | 761.00(580.97 to 976.28) | 120.88 | 433.69(387.85 to 479.75) | 894.27(707.99 to 1124.94) | 106.20 |
| Peru | 68.41(48.45 to 91.70) | 109.85(79.04 to 148.21) | 60.59 | 574.45(507.55 to 650.68) | 493.57(364.28 to 646.51) | -14.08 | 642.85(572.77 to 723.69) | 603.42(470.40 to 766.59) | -6.13 |
| Romania | 60.03(42.74 to 79.93) | 93.03(66.34 to 123.54) | 54.96 | 256.93(247.24 to 266.20) | 166.65(137.41 to 200.16) | -35.14 | 316.96(297.17 to 337.87) | 259.68(220.58 to 304.53) | -18.07 |
| Russian Federation | 76.78(54.61 to 101.50) | 87.87(62.41 to 115.74) | 14.44 | 175.25(171.56 to 181.86) | 125.91(110.17 to 142.45) | -28.15 | 252.03(229.25 to 277.76) | 213.78(183.68 to 245.92) | -15.18 |
| Saint Lucia | 140.15(100.28 to 184.82) | 196.70(139.85 to 261.85) | 40.36 | 734.52(680.83 to 793.42) | 801.79(666.74 to 959.66) | 9.16 | 874.66(804.64 to 947.94) | 998.50(856.75 to 1165.82) | 14.16 |
| Saint Vincent and the Grenadines | 137.54(98.92 to 184.33) | 200.45(140.85 to 264.67) | 45.74 | 606.95(545.99 to 662.98) | 860.62(736.24 to 1003.83) | 41.79 | 744.49(675.84 to 813.65) | 1061.06(917.51 to 1225.69) | 42.52 |
| Samoa | 158.34(115.44 to 209.99) | 221.94(159.30 to 298.29) | 40.16 | 896.80(688.53 to 1154.69) | 1040.74(819.34 to 1318.81) | 16.05 | 1055.14(831.73 to 1318.65) | 1262.68(1021.20 to 1546.64) | 19.67 |
| Serbia | 62.53(44.78 to 82.61) | 82.48(59.30 to 109.50) | 31.90 | 297.58(271.02 to 340.69) | 276.81(224.44 to 335.57) | -6.98 | 360.11(326.07 to 407.74) | 359.29(300.31 to 423.70) | -0.23 |
| South Africa | 88.20(63.18 to 116.62) | 117.19(84.02 to 153.71) | 32.86 | 547.47(506.13 to 600.69) | 647.92(599.90 to 695.18) | 18.35 | 635.68(585.91 to 693.49) | 765.11(706.48 to 825.38) | 20.36 |
| Sri Lanka | 108.53(77.79 to 141.99) | 165.95(118.46 to 219.14) | 52.91 | 578.97(513.33 to 637.77) | 513.39(382.40 to 675.79) | -11.33 | 687.49(615.57 to 755.03) | 679.34(540.26 to 854.06) | -1.19 |
| Suriname | 134.61(96.04 to 179.07) | 204.53(147.36 to 274.94) | 51.94 | 724.80(644.33 to 791.22) | 1091.68(900.71 to 1301.29) | 50.62 | 859.41(758.62 to 940.17) | 1296.21(1108.67 to 1518.53) | 50.83 |
| Thailand | 134.84(97.97 to 183.17) | 162.38(119.28 to 214.77) | 20.42 | 700.85(616.78 to 796.41) | 585.13(442.87 to 760.61) | -16.51 | 835.70(742.76 to 942.81) | 747.51(601.84 to 934.74) | -10.55 |
| Tonga | 158.29(112.94 to 207.59) | 217.97(156.40 to 288.48) | 37.70 | 662.20(530.71 to 887.08) | 947.14(725.06 to 1283.01) | 43.03 | 820.49(678.28 to 1046.37) | 1165.11(929.05 to 1507.65) | 42.00 |
| Turkey | 95.17(69.92 to 125.59) | 141.76(102.53 to 187.18) | 48.95 | 758.74(631.14 to 987.24) | 420.26(341.30 to 513.64) | -44.61 | 853.91(725.99 to 1083.24) | 562.02(473.61 to 667.56) | -34.18 |
| Turkmenistan | 80.81(57.27 to 107.19) | 115.25(82.07 to 153.23) | 42.62 | 443.35(415.05 to 472.18) | 536.17(434.62 to 674.11) | 20.94 | 524.16(486.76 to 564.92) | 651.42(540.11 to 791.75) | 24.28 |
| Tuvalu | 141.02(101.44 to 183.87) | 207.06(148.55 to 274.36) | 46.84 | 1012.82(801.60 to 1288.58) | 1181.01(850.20 to 1642.65) | 16.61 | 1153.84(934.40 to 1432.20) | 1388.07(1054.44 to 1852.50) | 20.30 |
| Venezuela (Bolivarian Republic of) | 141.69(101.02 to 189.41) | 196.53(137.22 to 262.96) | 38.71 | 379.94(359.03 to 401.22) | 824.37(616.59 to 1082.04) | 116.97 | 521.63(474.03 to 575.36) | 1020.90(801.82 to 1297.67) | 95.71 |

*CKD* Chronic kidney disease, *YLD* Years lived with disability, *YLL* Years of life lost, *DALY* Disability-adjusted life-years, *LICs* Low-income countries, *Lower* *MICs* Lower-middle income countries, *Upper MICs* Upper-middle income countries.

Table S3 The AAPC in age-standardized rates of YLD, YLL, and DALY for CKD in 137 low-and middle-income countries,1990-2019

| country | Age-standardized YLD rate per 100,000 persons | |  | Age-standardized YLL rate per 100,000 persons | |  | Age-standardized DALY rate per 100,000 persons | |
| --- | --- | --- | --- | --- | --- | --- | --- | --- |
|  | AAPC | P-Value |  | AAPC | P-Value |  | AAPC | P-Value |
| **LICs** |  |  |  |  |  |  |  |  |
| Afghanistan | 1.4(1.6 to 1.3) | <0.001 |  | -0.6(-0.7 to -0.6) | <0.001 |  | -0.4(-0.4 to -0.5) | <0.001 |
| Benin | 1.1(1.2 to 1.0) | <0.001 |  | -0.3(-0.4 to -0.2) | <0.001 |  | -0.2(-0.1 to -0.2) | <0.001 |
| Burkina Faso | 1.4(1.5 to 1.3) | <0.001 |  | 0.0(-0.1 to 0.2) | 0.493 |  | 0.2(0.3 to 0.0) | 0.010 |
| Burundi | 0.7(0.8 to 0.6) | <0.001 |  | -1.4(-1.5 to -1.2) | <0.001 |  | -1.2(-1.1 to -1.3) | <0.001 |
| Central African Republic | 1.0(1.1 to 0.9) | <0.001 |  | -0.1(-0.2 to 0.1) | 0.289 |  | 0.0(0.1 to -0.1) | 0.983 |
| Chad | 1.0(1.1 to 0.9) | <0.001 |  | 0.0(-0.1 to 0.1) | 0.713 |  | 0.1(0.2 to 0.0) | 0.222 |
| Democratic People's Republic of Korea | 0.5(0.6 to 0.4) | <0.001 |  | -0.5(-0.6 to -0.4) | <0.001 |  | -0.3(-0.2 to -0.4) | <0.001 |
| Democratic Republic of the Congo | 0.6(0.8 to 0.4) | <0.001 |  | -1.0(-1.0 to -0.9) | <0.001 |  | -0.8(-0.8 to -0.9) | <0.001 |
| Eritrea | 1.1(1.2 to 1.0) | <0.001 |  | -0.1(-0.2 to 0.1) | 0.358 |  | 0.0(0.2 to -0.1) | 0.615 |
| Ethiopia | 0.7(0.8 to 0.6) | <0.001 |  | -2.4(-2.5 to -2.3) | <0.001 |  | -2.2(-2.1 to -2.3) | <0.001 |
| Gambia | 1.1(1.1 to 1.0) | <0.001 |  | 0.0(-0.2 to 0.2) | 0.967 |  | 0.1(0.3 to -0.1) | 0.242 |
| Guinea | 1.0(1.1 to 0.9) | <0.001 |  | -0.2(-0.3 to -0.1) | 0.002 |  | -0.1(0.0 to -0.2) | 0.070 |
| Guinea-Bissau | 0.9(1.0 to 0.9) | <0.001 |  | -0.9(-0.9 to -0.8) | <0.001 |  | -0.8(-0.7 to -0.8) | <0.001 |
| Haiti | 1.5(1.6 to 1.3) | <0.001 |  | 0.1(-0.1 to 0.3) | 0.428 |  | 0.3(0.4 to 0.1) | 0.003 |
| Liberia | 1.0(1.1 to 0.9) | <0.001 |  | -0.9(-1.2 to -0.5) | <0.001 |  | -0.7(-0.4 to -1.1) | <0.001 |
| Madagascar | 0.7(0.8 to 0.6) | <0.001 |  | -0.8(-0.8 to -0.7) | <0.001 |  | -0.6(-0.6 to -0.7) | <0.001 |
| Malawi | 1.0(1.1 to 0.9) | <0.001 |  | -0.6(-0.8 to -0.4) | <0.001 |  | -0.5(-0.3 to -0.6) | <0.001 |
| Mali | 0.9(1.0 to 0.9) | <0.001 |  | -0.7(-0.9 to -0.5) | <0.001 |  | -0.6(-0.4 to -0.8) | <0.001 |
| Mozambique | 1.3(1.4 to 1.3) | <0.001 |  | 0.5(0.4 to 0.6) | <0.001 |  | 0.6(0.7 to 0.5) | <0.001 |
| Nepal | 1.0(1.3 to 0.7) | <0.001 |  | 0.7(0.4 to 1.1) | <0.001 |  | 0.8(1.1 to 0.5) | <0.001 |
| Niger | 0.7(0.8 to 0.7) | <0.001 |  | -1.0(-1.2 to -0.9) | <0.001 |  | -0.9(-0.8 to -1.0) | <0.001 |
| Rwanda | 1.0(1.1 to 0.8) | <0.001 |  | -2.3(-2.6 to -2.0) | <0.001 |  | -2.0(-1.7 to -2.3) | <0.001 |
| Sierra Leone | 1.1(1.2 to 1.0) | <0.001 |  | -0.3(-0.4 to -0.2) | <0.001 |  | -0.2(-0.1 to -0.3) | 0.001 |
| Somalia | 0.8(0.9 to 0.7) | <0.001 |  | -0.2(-0.2 to -0.1) | <0.001 |  | -0.1(0.0 to -0.1) | <0.001 |
| South Sudan | 0.9(0.9 to 0.8) | <0.001 |  | -0.3(-0.4 to -0.2) | <0.001 |  | -0.2(-0.1 to -0.3) | <0.001 |
| Syrian Arab Republic | 1.0(1.2 to 0.9) | <0.001 |  | -2.2(-2.6 to -1.8) | <0.001 |  | -1.7(-1.4 to -2.0) | <0.001 |
| Tajikistan | 1.4(1.7 to 1.2) | <0.001 |  | 2.7(2.3 to 3.1) | <0.001 |  | 2.3(2.6 to 2.0) | <0.001 |
| Togo | 1.0(1.1 to 0.9) | <0.001 |  | -0.5(-0.5 to -0.4) | <0.001 |  | -0.4(-0.3 to -0.4) | <0.001 |
| Uganda | 1.4(1.7 to 1.2) | <0.001 |  | -0.3(-0.6 to -0.1) | 0.002 |  | -0.2(0.0 to -0.3) | 0.054 |
| United Republic of Tanzania | 1.3(1.5 to 1.1) | <0.001 |  | 0.0(-0.1 to 0.1) | 0.679 |  | 0.1(0.2 to 0.0) | 0.008 |
| Yemen | 1.6(1.7 to 1.5) | <0.001 |  | -0.6(-0.7 to -0.5) | <0.001 |  | -0.2(-0.1 to -0.3) | 0.001 |
| **Lower MICs** |  |  |  |  |  |  |  |  |
| Angola | 1.3(1.5 to 1.2) | <0.001 |  | -0.8(-0.9 to -0.7) | <0.001 |  | -0.6(-0.6 to -0.7) | <0.001 |
| Bangladesh | 2.1(2.3 to 1.9) | <0.001 |  | -1.0(-1.3 to -0.7) | <0.001 |  | -0.4(-0.2 to -0.7) | 0.002 |
| Bhutan | 1.5(1.6 to 1.4) | <0.001 |  | 0.3(0.2 to 0.3) | <0.001 |  | 0.5(0.5 to 0.4) | <0.001 |
| Bolivia (Plurinational State of) | 1.1(1.3 to 1.0) | <0.001 |  | 0.6(0.6 to 0.7) | <0.001 |  | 0.7(0.8 to 0.6) | <0.001 |
| Cabo Verde | 1.6(1.7 to 1.5) | <0.001 |  | 0.3(0.0 to 0.5) | 0.047 |  | 0.4(0.7 to 0.2) | <0.001 |
| Cambodia | 1.2(1.2 to 1.1) | <0.001 |  | -1.3(-1.3 to -1.2) | <0.001 |  | -0.9(-0.8 to -1.0) | <0.001 |
| Cameroon | 1.5(1.6 to 1.4) | <0.001 |  | -0.3(-0.4 to -0.2) | <0.001 |  | -0.1(0.0 to -0.2) | 0.003 |
| Comoros | 0.9(0.9 to 0.8) | <0.001 |  | -0.7(-1.0 to -0.5) | <0.001 |  | -0.6(-0.4 to -0.8) | <0.001 |
| Congo | 1.1(1.2 to 1.0) | <0.001 |  | -1.1(-1.3 to -1.0) | <0.001 |  | -1.0(-0.8 to -1.1) | <0.001 |
| Côte d'Ivoire | 1.0(1.1 to 0.9) | <0.001 |  | -0.7(-0.9 to -0.6) | <0.001 |  | -0.6(-0.4 to -0.7) | <0.001 |
| Djibouti | 1.3(1.5 to 1.2) | <0.001 |  | 0.3(0.2 to 0.5) | <0.001 |  | 0.5(0.6 to 0.3) | <0.001 |
| Egypt | 1.6(1.7 to 1.5) | <0.001 |  | 0.3(0.2 to 0.4) | <0.001 |  | 0.5(0.6 to 0.4) | <0.001 |
| El Salvador | 2.8(3.4 to 2.2) | <0.001 |  | 4.2(3.5 to 4.9) | <0.001 |  | 4.0(4.6 to 3.3) | <0.001 |
| Eswatini | 1.1(1.3 to 0.8) | <0.001 |  | 1.4(0.7 to 2.1) | <0.001 |  | 1.4(2.0 to 0.8) | <0.001 |
| Ghana | 1.4(1.5 to 1.3) | <0.001 |  | 0.4(0.3 to 0.6) | <0.001 |  | 0.5(0.7 to 0.4) | <0.001 |
| Honduras | 1.1(1.1 to 1.0) | <0.001 |  | 2.5(2.3 to 2.8) | <0.001 |  | 2.3(2.5 to 2.1) | <0.001 |
| India | 0.5(0.8 to 0.2) | 0.001 |  | -0.5(-0.7 to -0.3) | <0.001 |  | -0.3(-0.1 to -0.5) | 0.003 |
| Indonesia | 1.1(1.2 to 1.1) | <0.001 |  | -0.4(-0.5 to -0.4) | <0.001 |  | -0.2(-0.1 to -0.3) | <0.001 |
| Kenya | 0.9(1.0 to 0.8) | <0.001 |  | 0.7(0.6 to 0.9) | <0.001 |  | 0.8(0.9 to 0.6) | <0.001 |
| Kiribati | 1.0(1.1 to 1.0) | <0.001 |  | 0.4(0.0 to 0.8) | 0.073 |  | 0.5(0.9 to 0.1) | 0.020 |
| Kyrgyzstan | 0.7(0.9 to 0.6) | <0.001 |  | -2.1(-2.6 to -1.6) | <0.001 |  | -1.6(-1.2 to -2.0) | <0.001 |
| Lao People's Democratic Republic | 1.2(1.3 to 1.1) | <0.001 |  | -1.3(-1.4 to -1.2) | <0.001 |  | -1.0(-1.0 to -1.1) | <0.001 |
| Lesotho | 1.2(1.4 to 1.0) | <0.001 |  | 3.2(2.8 to 3.5) | <0.001 |  | 3.0(3.3 to 2.7) | <0.001 |
| Mauritania | 0.9(1.0 to 0.9) | <0.001 |  | -1.8(-1.9 to -1.7) | <0.001 |  | -1.5(-1.4 to -1.6) | <0.001 |
| Micronesia (Federated States of) | 1.5(1.6 to 1.4) | <0.001 |  | 1.3(0.9 to 1.7) | <0.001 |  | 1.3(1.7 to 1.0) | <0.001 |
| Mongolia | 0.7(0.9 to 0.6) | <0.001 |  | -3.9(-4.4 to -3.4) | <0.001 |  | -3.3(-2.9 to -3.8) | <0.001 |
| Morocco | 2.7(2.8 to 2.5) | <0.001 |  | 0.4(0.3 to 0.5) | <0.001 |  | 0.8(0.8 to 0.7) | <0.001 |
| Myanmar | 1.2(1.3 to 1.1) | <0.001 |  | -1.1(-1.3 to -0.9) | <0.001 |  | -0.8(-0.6 to -0.9) | <0.001 |
| Nicaragua | 1.3(1.5 to 1.1) | <0.001 |  | 2.9(2.5 to 3.2) | <0.001 |  | 2.7(2.9 to 2.4) | <0.001 |
| Nigeria | 1.3(1.4 to 1.1) | <0.001 |  | -0.6(-0.7 to -0.6) | <0.001 |  | -0.4(-0.4 to -0.5) | <0.001 |
| Pakistan | 1.2(1.3 to 1.1) | <0.001 |  | 1.5(1.2 to 1.8) | <0.001 |  | 1.5(1.7 to 1.2) | <0.001 |
| Palestine | 1.0(1.1 to 1.0) | <0.001 |  | -1.1(-1.4 to -0.9) | <0.001 |  | -0.8(-0.6 to -1.0) | <0.001 |
| Papua New Guinea | 1.0(1.0 to 0.9) | <0.001 |  | 0.4(0.2 to 0.6) | <0.001 |  | 0.5(0.6 to 0.4) | <0.001 |
| Philippines | 1.4(1.5 to 1.4) | <0.001 |  | 1.3(1.0 to 1.6) | <0.001 |  | 1.3(1.6 to 1.1) | <0.001 |
| Republic of Moldova | 1.0(1.2 to 0.8) | <0.001 |  | 1.8(1.3 to 2.3) | <0.001 |  | 1.5(1.9 to 1.2) | <0.001 |
| Sao Tome and Principe | 1.2(1.3 to 1.1) | <0.001 |  | 0.1(-0.1 to 0.3) | 0.333 |  | 0.2(0.4 to 0.0) | 0.053 |
| Senegal | 0.5(0.6 to 0.4) | <0.001 |  | -0.7(-0.9 to -0.5) | <0.001 |  | -0.6(-0.4 to -0.8) | <0.001 |
| Solomon Islands | 0.6(0.6 to 0.5) | <0.001 |  | -2.0(-2.4 to -1.6) | <0.001 |  | -1.6(-1.3 to -2.0) | <0.001 |
| Sudan | 1.7(1.8 to 1.7) | <0.001 |  | -0.3(-0.4 to -0.2) | <0.001 |  | 0.0(0.1 to -0.1) | 0.796 |
| Timor-Leste | 1.3(1.4 to 1.2) | <0.001 |  | -0.5(-0.8 to -0.2) | 0.001 |  | -0.2(0.0 to -0.5) | 0.046 |
| Tunisia | 1.5(1.6 to 1.4) | <0.001 |  | -0.5(-0.5 to -0.4) | <0.001 |  | -0.1(0.0 to -0.1) | <0.001 |
| Ukraine | 1.1(1.3 to 0.9) | <0.001 |  | 3.8(3.2 to 4.3) | <0.001 |  | 2.6(3.0 to 2.3) | <0.001 |
| Uzbekistan | 1.1(1.3 to 1.0) | <0.001 |  | 0.1(-0.7 to 0.8) | 0.892 |  | 0.2(0.9 to -0.4) | 0.463 |
| Vanuatu | 1.2(1.3 to 1.2) | <0.001 |  | 1.9(1.7 to 2.0) | <0.001 |  | 1.8(1.9 to 1.7) | <0.001 |
| Viet Nam | 1.3(1.5 to 1.1) | <0.001 |  | -0.6(-1.1 to -0.1) | 0.017 |  | -0.3(0.1 to -0.7) | 0.122 |
| Zambia | 1.3(1.4 to 1.2) | <0.001 |  | -0.7(-0.9 to -0.5) | <0.001 |  | -0.5(-0.4 to -0.7) | <0.001 |
| Zimbabwe | 0.8(1.1 to 0.5) | <0.001 |  | 1.8(1.4 to 2.2) | <0.001 |  | 1.7(2.0 to 1.4) | <0.001 |
| **Upper MICs** |  |  |  |  |  |  |  |  |
| Albania | 0.9(1.0 to 0.9) | <0.001 |  | -1.3(-1.6 to -1.1) | <0.001 |  | -0.8(-0.6 to -1.0) | <0.001 |
| Algeria | 1.5(1.5 to 1.5) | <0.001 |  | -0.9(-1.1 to -0.7) | <0.001 |  | -0.5(-0.3 to -0.6) | <0.001 |
| American Samoa | 1.4(1.5 to 1.3) | <0.001 |  | 1.5(1.3 to 1.6) | <0.001 |  | 1.5(1.6 to 1.3) | <0.001 |
| Argentina | 0.5(0.6 to 0.4) | <0.001 |  | 0.1(-0.2 to 0.4) | 0.506 |  | 0.2(0.4 to -0.1) | 0.218 |
| Armenia | 1.6(1.8 to 1.5) | <0.001 |  | 4.6(4.1 to 5.1) | <0.001 |  | 3.6(3.8 to 3.3) | <0.001 |
| Azerbaijan | 1.6(1.7 to 1.5) | <0.001 |  | 1.2(0.8 to 1.6) | <0.001 |  | 1.3(1.6 to 0.9) | <0.001 |
| Belarus | 0.9(1.2 to 0.6) | <0.001 |  | 2.4(1.5 to 3.4) | <0.001 |  | 1.8(2.3 to 1.3) | <0.001 |
| Belize | 1.6(1.7 to 1.5) | <0.001 |  | 2.2(1.8 to 2.7) | <0.001 |  | 2.1(2.5 to 1.7) | <0.001 |
| Bosnia and Herzegovina | 1.4(1.5 to 1.3) | <0.001 |  | 0.0(-0.2 to 0.2) | 0.959 |  | 0.4(0.5 to 0.2) | <0.001 |
| Botswana | 1.4(1.6 to 1.3) | <0.001 |  | 0.5(0.1 to 0.9) | 0.028 |  | 0.6(1.0 to 0.2) | 0.004 |
| Brazil | 0.2(0.3 to 0.1) | 0.004 |  | -0.6(-0.7 to -0.6) | <0.001 |  | -0.5(-0.4 to -0.6) | <0.001 |
| Bulgaria | 1.4(1.5 to 1.4) | <0.001 |  | 2.4(2.0 to 2.7) | <0.001 |  | 2.2(2.4 to 1.9) | <0.001 |
| China | 1.3(1.7 to 1.0) | <0.001 |  | -1.2(-1.3 to -1.1) | <0.001 |  | -0.6(-0.5 to -0.7) | <0.001 |
| Colombia | 0.6(0.7 to 0.5) | <0.001 |  | -1.1(-1.3 to -1.0) | <0.001 |  | -0.7(-0.6 to -0.8) | <0.001 |
| Costa Rica | 0.3(0.4 to 0.2) | <0.001 |  | 1.8(1.3 to 2.4) | <0.001 |  | 1.3(1.7 to 1.0) | <0.001 |
| Cuba | 1.3(1.3 to 1.2) | <0.001 |  | 2.0(1.8 to 2.2) | <0.001 |  | 1.8(2.0 to 1.6) | <0.001 |
| Dominica | 1.0(1.2 to 0.9) | <0.001 |  | 1.3(1.1 to 1.4) | <0.001 |  | 1.2(1.3 to 1.1) | <0.001 |
| Dominican Republic | 1.5(1.5 to 1.4) | <0.001 |  | 2.5(2.2 to 2.8) | <0.001 |  | 2.3(2.5 to 2.0) | <0.001 |
| Ecuador | 1.9(2.0 to 1.8) | <0.001 |  | 2.6(1.9 to 3.3) | <0.001 |  | 2.5(3.1 to 1.9) | <0.001 |
| Equatorial Guinea | 2.0(2.1 to 1.8) | <0.001 |  | -0.4(-0.6 to -0.2) | 0.001 |  | -0.2(0.0 to -0.4) | 0.045 |
| Fiji | 1.0(1.1 to 1.0) | <0.001 |  | 0.4(0.0 to 0.7) | 0.069 |  | 0.5(0.8 to 0.1) | 0.008 |
| Gabon | 1.4(1.5 to 1.4) | <0.001 |  | 0.0(-0.2 to 0.2) | 0.880 |  | 0.1(0.3 to -0.1) | 0.195 |
| Georgia | 1.4(1.5 to 1.3) | <0.001 |  | 3.1(2.3 to 3.9) | <0.001 |  | 2.6(3.3 to 2.0) | <0.001 |
| Grenada | 1.5(1.6 to 1.3) | <0.001 |  | 1.4(1.2 to 1.6) | <0.001 |  | 1.4(1.5 to 1.2) | <0.001 |
| Guatemala | 1.8(1.9 to 1.6) | <0.001 |  | 2.6(2.1 to 3.1) | <0.001 |  | 2.5(2.9 to 2.1) | <0.001 |
| Guyana | 1.4(1.5 to 1.3) | <0.001 |  | 2.5(2.2 to 2.7) | <0.001 |  | 2.3(2.5 to 2.1) | <0.001 |
| Iran (Islamic Republic of) | 0.4(0.5 to 0.3) | <0.001 |  | -1.4(-1.4 to -1.3) | <0.001 |  | -1.0(-0.9 to -1.1) | <0.001 |
| Iraq | 1.4(1.5 to 1.4) | <0.001 |  | -0.9(-0.9 to -0.8) | <0.001 |  | -0.5(-0.5 to -0.5) | <0.001 |
| Jamaica | 1.2(1.4 to 1.1) | <0.001 |  | 0.8(0.2 to 1.4) | 0.011 |  | 0.9(1.3 to 0.4) | 0.001 |
| Jordan | 1.4(1.5 to 1.4) | <0.001 |  | -0.6(-0.7 to -0.4) | <0.001 |  | -0.2(-0.1 to -0.3) | 0.001 |
| Kazakhstan | 1.3(1.5 to 1.1) | <0.001 |  | -0.3(-0.8 to 0.1) | 0.154 |  | 0.0(0.4 to -0.3) | 0.925 |
| Lebanon | 1.5(1.5 to 1.4) | <0.001 |  | -1.4(-1.6 to -1.2) | <0.001 |  | -0.8(-0.6 to -1.0) | <0.001 |
| Libya | 1.4(1.5 to 1.3) | <0.001 |  | 0.1(-0.1 to 0.3) | 0.168 |  | 0.4(0.5 to 0.2) | <0.001 |
| Malaysia | 1.7(1.9 to 1.6) | <0.001 |  | -0.8(-1.0 to -0.6) | <0.001 |  | -0.3(-0.1 to -0.4) | 0.001 |
| Maldives | 1.1(1.2 to 1.0) | <0.001 |  | -2.9(-3.2 to -2.5) | <0.001 |  | -2.3(-2.0 to -2.6) | <0.001 |
| Marshall Islands | 1.2(1.2 to 1.2) | <0.001 |  | 1.0(0.7 to 1.3) | <0.001 |  | 1.0(1.3 to 0.8) | <0.001 |
| Mauritius | 1.8(1.9 to 1.7) | <0.001 |  | 2.1(1.7 to 2.5) | <0.001 |  | 2.1(2.4 to 1.7) | <0.001 |
| Mexico | 1.7(1.8 to 1.6) | <0.001 |  | 3.1(2.6 to 3.7) | <0.001 |  | 2.9(3.3 to 2.4) | <0.001 |
| Montenegro | 0.9(1.0 to 0.9) | <0.001 |  | 0.0(-0.2 to 0.2) | 0.957 |  | 0.2(0.4 to 0.0) | 0.031 |
| Namibia | 1.0(1.2 to 0.7) | <0.001 |  | -0.6(-1.0 to -0.1) | 0.011 |  | -0.4(0.0 to -0.8) | 0.034 |
| Nauru | 1.2(1.3 to 1.1) | <0.001 |  | 0.9(0.5 to 1.3) | <0.001 |  | 0.9(1.3 to 0.6) | <0.001 |
| North Macedonia | 1.4(1.6 to 1.2) | <0.001 |  | -0.4(-0.6 to -0.1) | 0.001 |  | 0.1(0.3 to -0.1) | 0.436 |
| Paraguay | 1.3(1.4 to 1.3) | <0.001 |  | 3.3(3.1 to 3.6) | <0.001 |  | 3.0(3.2 to 2.8) | <0.001 |
| Peru | 1.7(1.8 to 1.6) | <0.001 |  | -0.4(-0.7 to -0.2) | <0.001 |  | -0.1(0.0 to -0.3) | 0.094 |
| Romania | 1.9(2.2 to 1.6) | <0.001 |  | -1.2(-1.7 to -0.8) | <0.001 |  | -0.4(0.1 to -0.8) | 0.104 |
| Russian Federation | 0.6(0.7 to 0.6) | <0.001 |  | -2.2(-2.6 to -1.7) | <0.001 |  | -1.3(-1.0 to -1.5) | <0.001 |
| Saint Lucia | 1.1(1.2 to 0.9) | <0.001 |  | 0.5(0.3 to 0.7) | <0.001 |  | 0.6(0.8 to 0.5) | <0.001 |
| Saint Vincent and the Grenadines | 1.3(1.4 to 1.1) | <0.001 |  | 1.4(1.3 to 1.6) | <0.001 |  | 1.4(1.5 to 1.3) | <0.001 |
| Samoa | 1.1(1.2 to 1.0) | <0.001 |  | 0.4(0.2 to 0.7) | <0.001 |  | 0.5(0.8 to 0.3) | <0.001 |
| Serbia | 1.1(1.1 to 1.0) | <0.001 |  | -0.2(-0.3 to -0.1) | 0.007 |  | 0.1(0.2 to -0.1) | 0.382 |
| South Africa | 1.0(1.1 to 0.8) | <0.001 |  | 0.7(0.3 to 1.1) | 0.001 |  | 0.7(1.1 to 0.4) | <0.001 |
| Sri Lanka | 1.6(1.6 to 1.5) | <0.001 |  | -0.3(-0.6 to 0.0) | 0.066 |  | 0.1(0.3 to -0.2) | 0.522 |
| Suriname | 1.4(1.5 to 1.4) | <0.001 |  | 1.4(1.1 to 1.7) | <0.001 |  | 1.4(1.6 to 1.2) | <0.001 |
| Thailand | 0.9(1.1 to 0.8) | <0.001 |  | -1.1(-1.4 to -0.9) | <0.001 |  | -0.8(-0.6 to -0.9) | <0.001 |
| Tonga | 1.1(1.1 to 1.1) | <0.001 |  | 1.2(0.8 to 1.6) | <0.001 |  | 1.2(1.5 to 0.8) | <0.001 |
| Turkey | 1.7(2.1 to 1.3) | <0.001 |  | -2.1(-2.4 to -1.8) | <0.001 |  | -1.4(-1.2 to -1.6) | <0.001 |
| Turkmenistan | 1.4(1.5 to 1.3) | <0.001 |  | 0.2(-0.1 to 0.5) | 0.221 |  | 0.4(0.6 to 0.1) | 0.004 |
| Tuvalu | 1.3(1.3 to 1.2) | <0.001 |  | 0.5(0.3 to 0.7) | <0.001 |  | 0.6(0.8 to 0.5) | <0.001 |
| Venezuela (Bolivarian Republic of) | 1.1(1.2 to 1.0) | <0.001 |  | 2.1(1.6 to 2.7) | <0.001 |  | 1.9(2.3 to 1.5) | <0.001 |

*CKD* Chronic kidney disease, *AAPC* average annual percentage change, *YLD* Years lived with disability, *YLL* Years of life lost, *DALY* Disability-adjusted life-years, *LICs* Low-income countries, *Lower* *MICs* Lower-middle income countries, *Upper MICs* Upper-middle income countries.
